# Inter‐Laboratory Validation of Nodal/Paranodal Antibody Testing

**DOI:** 10.1111/jns.70000

**Published:** 2025-01-29

**Authors:** Cinta Lleixà, Maarten Titulaer, Sophia Rohrbacher, Victor Mgbachi, Susan Halstead, Janev Fehmi, Elba Pascual‐Goñi, Louisa Zhu, Luise Appeltshauser, Suzanne Franken, Manuela Paunovic, Patrick Waters, Hugh Willison, Claudia Sommer, Luis Querol, Ruth Huizinga, Kathrin Doppler, Simon Rinaldi

**Affiliations:** ^1^ Neuromuscular Diseases Unit Hospital de la Santa Creu i Sant Pau Barcelona Spain; ^2^ Centro Para la Investigación en Red en Enfermedades Raras (CIBERER) Madrid Spain; ^3^ Department of Neurology Erasmus MC University Medical Center Rotterdam Netherlands; ^4^ Department of Neurology University Hospital Würzburg Würzburg Germany; ^5^ Nuffield Department of Clinical Neurosciences University of Oxford Oxford UK; ^6^ School of Infection and Immunity University of Glasgow Glasgow UK; ^7^ Department of Immunology Erasmus MC University Medical Center Rotterdam Netherlands

**Keywords:** autoantibodies, CASPR1, contactin‐1, immunoassay, neurofascin

## Abstract

**Background and Aims:**

Reliable detection of antibodies against nodal targets is vital for the diagnosis of autoimmune nodopathies. The performance characteristics of recently developed in‐house assays are unknown. We compared testing at four centres.

**Methods:**

Each submitted 29–40 serum samples to a coordinating centre from one of three groups: (1) autoimmune nodopathy patients, with positive nodal/paranodal antibodies; (2) seronegative patients with other inflammatory neuropathies, and (3) healthy individuals or those with other neurological diseases. The coordinating centre recoded all samples and returned 160 identical aliquots to each testing centre for blinded testing. Once data from all centres had been received by the coordinating centre, unblinded results were returned for analysis. Sensitivity was defined by the proportion of group 1 samples returned as positive. Accuracy was defined as 0.075(sensitivity) + 0.925(specificity).

**Results:**

Centres performed various combinations of ELISA, cell‐based (CBAs) and teased‐nerve fibre assays. All labs produced highly accurate results (96%–100%) and concordance for the overall result across at least 3 or all 4 test centres was observed for 98% and 89% of the samples respectively. However, 10/30 individual assays (6/14 CBAs and 4/16 ELISAs) were less than 90% sensitive. Only 3 assays had more than 1 false positive result (2 ELISAs and 1 CBA). Combining different assay modalities to produce an overall result did not improve accuracy. Inter‐laboratory consistency in the determination of antibody subclasses was poor.

**Interpretation:**

Although most samples were correctly categorised in all 4 centres, the use of a specific test modality or multiple tests did not guarantee accuracy. Early and repeated interlaboratory testing with sharing of samples is important to understand test performance and reproducibility, identify areas for improvement and maintain consistency. To aid this, we provide detailed methods for the best performing tests. Further standardisation of antibody subclass determination is required.

## 1 Introduction

Autoimmune nodopathies are increasingly recognised as a group of clinically and pathologically distinct disorders [1, 2, 3, 4, 5]. The accurate detection of antibodies targeting nodal/paranodal cell adhesion molecules is essential for their diagnosis and optimal management, not least because the result has important treatment implications [6, 7]. Although patients with autoimmune nodopathies may have clinical features which overlap with those found in other inflammatory neuropathies, they frequently do not respond to the usual therapies used in the seronegative disorders, most notably intravenous immunoglobulin. Testing for paranodal/nodal antibodies (PNAb) typically involves the use of several assays evaluating multiple different antigens. The initial clinical associations for neurofascin‐155 (NF155), neurofascin‐140/186 (NF186), panNF, contactin‐1 (CNTN1) and contactin‐associated protein 1 (Caspr1) antibodies were established using transiently‐transfected cell‐based assays and ELISAs, often supported by binding patterns on teased‐nerve fibres or other tissue‐based assays [1, 2, 3, 4, 5]. However, the performance characteristics of these assays in routine diagnostic practice has not previously been evaluated. Furthermore, the intra‐laboratory reliability and inter‐laboratory consistency of PNAb testing have not been assessed. Previous studies evaluating ganglioside antibody testing showed considerable inter‐laboratory variability, particularly when different in‐house methods were used, which was partially reduced using standardised methods and reagents [8].

Live cell‐based assays (CBAs) have theoretical advantages for the detection of pathogenic antibodies, as the antigen of interest is displayed in its native conformation within the cell membrane. Fixing cells prior to serum incubation has the potential to mask epitopes and/or create neo‐epitopes, and may partially or completely expose irrelevant intracellular epitopes and other antigens for antibody binding. Similarly, in ELISA, when coating with full‐length protein, intracellular epitopes are likely exposed, and antigens may bind to the plate in a way which distorts their conformation and/or masks epitopes. However, ELISA has the advantages of not requiring cell‐culture facilities, is a higher throughput assay, and produces quantitative results. In contrast, cell‐based assays tend to be manually read and coarsely end‐point titred using fluorescence microscopy, although some centres use quantitative flow cytometry. Binding assays on fixed and permeabilized teased nerve fibres can be used to confirm the paranodal/nodal localization of the autoantigen [9]. This also allows simultaneous screening for multiple paranodal/nodal autoantigens, including the detection of unknown autoantigens. However, these assays are time‐consuming, require dissected nerve tissue, and are not specific for a particular autoantigen. The sensitivity and specificity of assay methods is likely to vary by antigen and between laboratories. Some previous studies have shown cell‐based assays to be more sensitive (and/or accurate) than ELISA [10], whereas others have shown the reverse [11].

In this study, we assess the performance of PNAb testing in four European test centres (TC1–4) where in‐house testing for PNAbs has been developed and is used routinely for patient care. We initially evaluate, and compare between centres, overall performance, before then exploring the characteristics of the individual assays on an antigen‐by‐antigen basis. Finally, we assess the consistency and reproducibility of IgG subclass determination.

## 2 Materials and Methods

Four European test centres (Barcelona, Oxford, Rotterdam and Würzburg) and one coordinating centre (Glasgow) participated. A study protocol (Appendix S1) for sample selection, grouping, submission, recoding, testing and analysis was agreed with input from all 5 centres.

### 2.1 Sample Selection and Group Definitions

Each laboratory was asked to submit 300 μL of 25–45 serum samples to the coordinating centre. These were drawn from 3 pre‐defined groups;

1. Seropositive patients with treatment‐responsive autoimmune nodopathies (i.e., PNAb positive at the submitting centre and with improvement in disability following immunotherapy), defined as an improvement in one or more of MRC sum‐score (by ≥ 2/70), inflammatory neuropathy Rasch‐built overall disability scale (≥ 6% or 4/48), modified Rankin scale (≥ 1), Guillain‐Barré syndrome Disability Score (≥ 1) and/or overall neuropathy limitations scale (≥ 1).
2. Seronegative patients with potentially overlapping clinical phenotypes—previously PNAb negative at the submitting centre and meeting current diagnostic criteria for one of the other types of inflammatory neuropathy (predominantly chronic inflammatory demyelinating polyneuropathy, CIDP [12], or Guillain‐Barré syndrome, GBS [13]).
3. Patients with unrelated diseases or healthy controls.

In the absence of a ‘gold standard’ for the diagnosis of autoimmune nodopathies, improvement following immunotherapy was included in the criteria to reduce the chance of including false positive sera within group 1. Groups 2 and 3 acted as controls fulfilling slightly different functions. Group 2 represents the population likely to form the vast majority of negative results in routine diagnostic testing. Group 3 is a cohort in which any positive result can be confidentially assigned as false positive/clinically irrelevant.

### 2.2 Double Aliquots

Each centre had the option to provide a larger volume (600 μL) of some samples to allow double aliquoting. On receipt of the submitted samples, the coordinating centre decided which of the sera with larger volumes to divide into two equal halves. All individual sera and duplicated pairs were then further divided into five identical aliquots of 60 μL. All samples were recoded uniquely, that is, the same sample had different codes at each centre and duplicates had unique codes. This approach ensured the study was fully blinded. One set was then sent to each testing centre, and one remained at the coordinating centre.

### 2.3 Testing Strategy

Each centre tested an identical, blinded set of serum samples using their usual protocols. These protocols were submitted to the co‐ordinating centre (see Table 1 for a summary of the testing approaches taken and how an overall result was decided at each centre, and Appendix S1 for assay method details). Results were then returned to the coordinating centre via email using a standardised report form. Each centre provided an overall positive or negative result for each sample along with results for each individual assay (e.g., CBA, ELISA, and teased nerve fibre). If subclass testing and end‐point titration were performed routinely, these results were also returned. Some centres subsequently performed follow‐up, unblinded testing of new assays or to explore reasons for discrepancies during the blinded testing period, but these results did not contribute to the accuracy evaluation or initial analyses.

**TABLE 1:** Nodal/paranodal antibody testing strategies prior to and during study.

Test centre	Summary of testing strategy					Comments	Strategy for combing multiple tests into overall result (see also Table A2)
	Primary assay	Secondary assay	Assay for IgG subclass and titre	Screening dilution/positivity threshold	Titration strategy		
TC1	Live CBA (NF155, NF186, CNTN1/Caspr1)	ELISA (all samples – NF155, NF186, CNTN1, Caspr1)	Live CBA	1:100 (NF155, NF186), 1:40 (CNTN1/Caspr) ELISA: corrected OD > 0.1	Doubling dilution from 1:100 to 1:6400	If CNTN1/Caspr1 positive on initial screening, a further live CBA is performed against CNTN1 and Caspr1 separately	Clear positives on live CBA reported as positive, borderline cases (1–2@1:100 only, or poor localisation) only reported as positive if ELISA also positive (corrected OD > 0.1 at 1:100)
TC2	Fixed CBA (NF155, NF140, NF186, CNTN1, CNTN1/Caspr1)	ELISA (NF155, NF186, CNTN1, Caspr1)	ELISA	1:100 ELISA: OD > 0.1 and > 2× blank	Tripling dilution from 1:100 to 1:24300	IHC with teased nerve fibres also performed for borderline cases only	Overall positive if positive on CBA and one other assay, or clear positive on CBA only
TC3	Live CBA (NF155) & Fixed CBA (CNTN1 and CNTN1/Caspr1)	Not performed	Not performed	1:100	N/A	All samples tested on both CNTN1 monotransfected and CNTN1/Caspr1 co‐transfected assays NF186/140 antibodies not assessed during blinded study	N/A
TC4	ELISA	—	ELISA	OD > 5SD above control mean at 1:100	Repeated at 1:500, 1:1000, 1:2000, 1:5000, 1:7500, 1:10000		N/A

*Note:* All assays were developed ‘in‐house’.

Abbreviations: Caspr1—CNTN associated protein; CBA—cell based assay; CNTN1—contactin‐1; CNTN1/Caspr1—indicates co‐transfection for CBA; ELISA—enzyme linked immunosorbent assay; IHC—immunohistochemistry; IHC—immunohistochemistry; NF—neurofascin; TC—test centre.

### 2.4 Analyses

Each centre provided an overall result for each serum. Sensitivity was based on group 1 samples (seropositive autoimmune nodopathy samples). Specificity was based on both group 2 (seronegative neurological disease) and group 3 samples (healthy control samples and other non‐neurological disease controls). Accuracy was defined as 0.075 (sensitivity) + 0.925 (specificity) based on the seropositivity rate of 7.5% observed over 3 years of routine testing in TC1. The performance of individual labs and assays and the utility of combining multiple tests to produce an overall result were examined and compared using McNemar's test. For this purpose, positive results for group 1 samples and negative results for group 2/3 samples were considered as correct. Spearman's co‐efficient was used to evaluate the correlation in end‐point titre between test centres.

**FIGURE 1:**
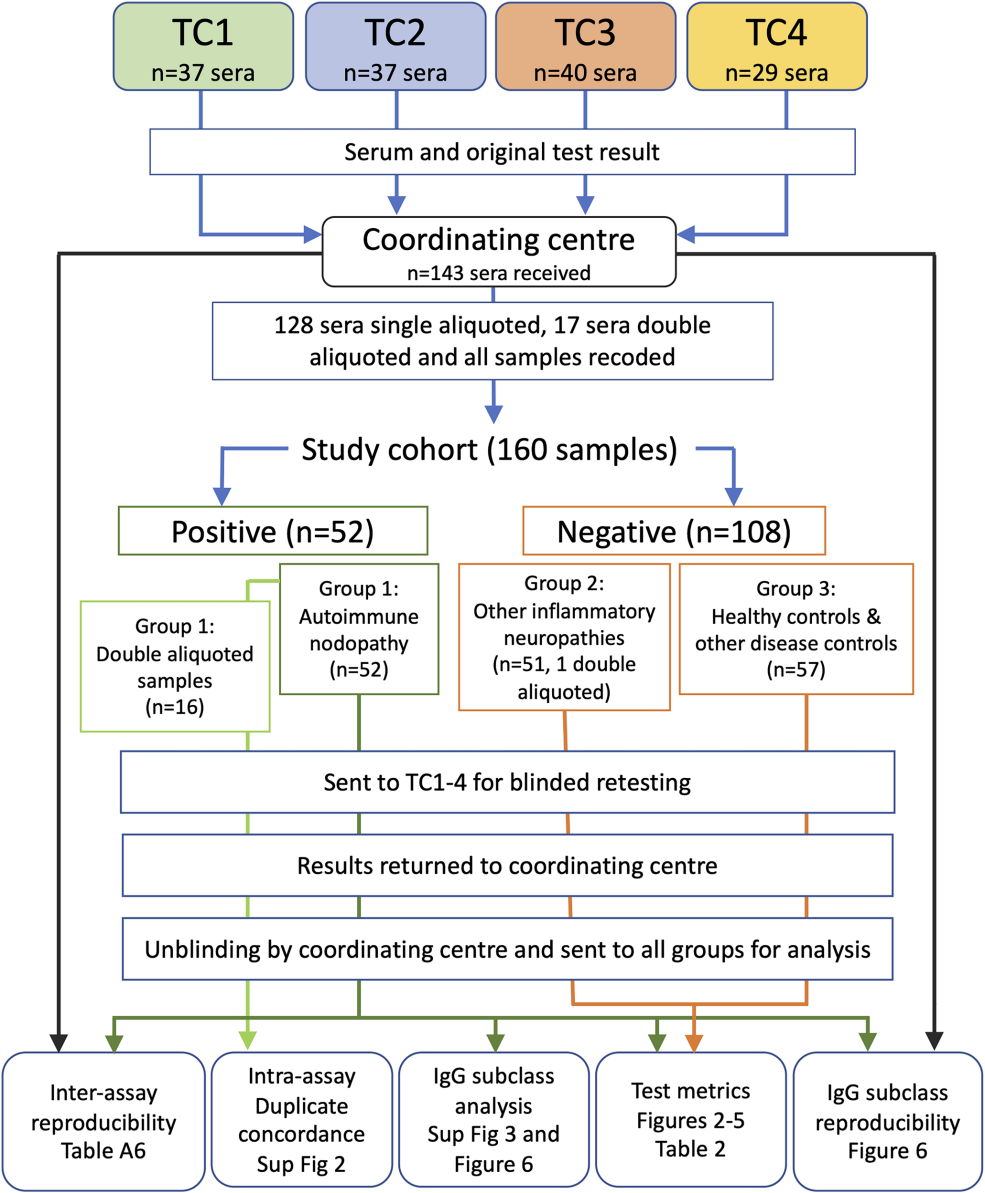
Study overview. Four testing centres (TC) provided 143 sera, 17 of which were double aliquoted to the coordinating centre. All samples were re‐coded uniquely for each centre and sent for testing. The cohort was tested blinded on 23 antibody assays. Each centre reported an overall result for each serum, results on individual tests and TC1, TC2 and TC4 reported the IgG subclasses on positive sera. When all results were reported to the coordinating centre the results were released to all groups for analysis.

To assess inter‐test reproducibility the blinded test results at each centre were compared with their previously submitted results. Short‐term/intra‐assay test reproducibility was examined by comparing the results of duplicate samples (see Figure 1 for study overview).

Some recent literature suggests identification of IgG subclasses of target specific antibodies is important in treatment decisions [14]. IgG subclasses were determined by 3 out of 4 centres (and, 1, 2, and 4) for all samples identified as seropositive. A simple matching coefficient (number of samples with agreement/total number of samples tested) was initially calculated for pairwise comparison of each IgG subclass between test centres. Further analysis examined the correlation of optical densities between subclass ELISAs across the two centres running these assays.

## 3 Results

### 3.1 Test Cohort

The test cohort consisted of 52 (‘group 1’) samples (from 36 patients) previously found to have antibodies to NF155 (17), NF186 (4), NF155 and NF186 (panNF, 8), CNTN1 (15) or Caspr1 (8), 51 (‘group 2’) disease controls negative for paranodal antibodies: CIDP (43), GBS (3), anti‐MAG neuropathy (3), multifocal motor neuropathy (MMN, 1), combined central and peripheral demyelination (CCPD, 1), and 57 (‘group 3’) control sera from healthy individuals (41), or patients with motor neuron disease (MND, 4), myasthenia gravis (3), genetic neuropathies (2) or non‐neurological conditions (7) (Table A1). All included samples had been previously collected between September 2002 and September 2021, first tested between November 2011 and August 2021, and stored at −80°C prior to use in this study. The number of previous freeze–thaw cycles was not recorded. A summary of the demographics, clinical features and treatment responses of the group 1 patients is given in Tables A2–A3.

### 3.2 Testing Strategies

Testing approaches varied between centres (Table 1), and some ran multiple assays for a single antigen. Different assays for the same antigen within the same lab were not always concordant, meaning some positive or negative results on individual tests were discounted in order to generate an overall result (Table A4).

### 3.3 Overall Results

Overall, the four laboratories generated concordant dichotomised results (positive or negative) in 143/160 (89%) samples: 39/52 (75%) group 1 antibody positive samples and 103/108 (95.4%) negative control samples. A single healthy sample was classified as NF155 positive at TC2 only, and 4 group 2 disease controls were called positive at single centres: two at TC2 for NF155, one at TC3 for contactin‐1 and one at TC4 for NF155. Although these samples were drawn from patients with some clinical features in keeping with autoimmune nodopathy, all 4 showed a good response to either IVIg or steroids, compared with only 8/34 in group 1 (*p* = 0.007, Fisher's exact test). All samples in Groups 2 and 3 were submitted as negative. None of these were positive at more than one site on blinded retesting, hence all 108 were deemed suitable as negative controls. As such, no samples were reclassified from one group to another following blinded retesting. One sample, initially collected and tested in February 2019, and submitted as seropositive for NF186 and double aliquoted, was only (NF155) positive on blinded testing with one aliquot at one centre only. As such, both aliquots were excluded from the accuracy analyses, but retained for concordance and reproducibility evaluations.

**FIGURE 2:**
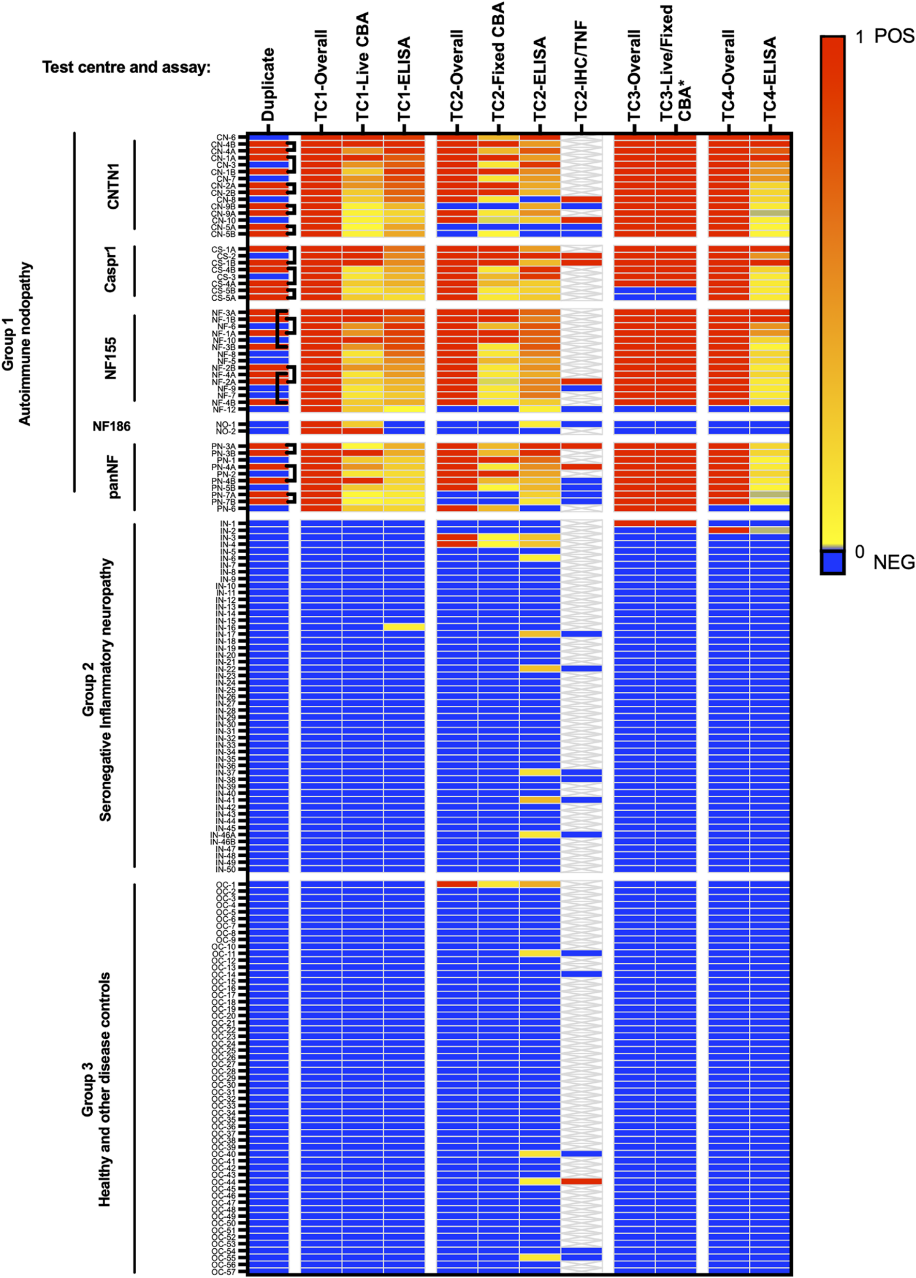
Heatmap of overall and assay type results by test centre. Group 1 samples are arranged from in descending order of titre for each antigen. The duplicate column indicates paired samples (red) linked by square brackets. The excluded paired sample is omitted from this figure. White boxes with a grey cross indicate test not performed with this sample. * Live CBA in TC3 only tests against NF155, fixed CBA only against CNTN1 and CNTN1/Caspr1. No samples were reclassified from one group to another following blinded retesting. Caspr1—contactin associated protein 1; CBA—cell‐based assay; CNTN1—contactin‐1; ELISA—enzyme linked immunosorbent assay; IHC/TNF—teased nerve fibre assay; NF—neurofascin.

Across a minimum of three sites, 47/50 (94%) of the remaining positive samples and all negative samples (108/108) were correctly classified by blinded retesting (155/158 [98%] in total) (Figure 2). Considering the overall result, and all antigens together, the performance of the four centres varied more in terms of sensitivity (84%–100%) than specificity (97%–100%) or accuracy (96%–100%) (Table 2).

**TABLE 2:** —Sensitivity, specificity, accuracy and consistency of overall results by test centre.

	TC1	TC2	TC3	TC4
	Number (%) returned as positive on blinded retesting			
Antibody Positive AIN (*n* = 50)^a^ (%)	50 (100)	42 (84)	45 (90)	46 (92)
Healthy Controls (*n* = 57) (%)	0	1 (2)	0	0
Disease controls (*n* = 51) (%)	0	2 (4)	1 (2)	1 (2)
Total Controls (*n* = 108) (%)	0	3 (3)	1 (1)	1 (1)
Sensitivity (%)	100	84	90	92
Specificity (%)	100	97	99	99
Accuracy (%)	100	96	98	99
Positive Likelihood Ratio (LR+)	∞	30	97	99
LR+ 95% CI^b^	14–3430	9.8–93	14–690	14–700
Negative Likelihood Ratio (LR−)	0	0.16	0.10	0.08
LR− 95% CI^b^	0.00–0.16	0.09‐0.31	0.04–0.23	0.03–0.21
Intra‐assay consistency (%)	100	100	94	94
Inter‐assay consistency (%)	100	96	100	94

Abbreviations: AIN—autoimmune nodopathy; CI—confidence interval; LR—likelihood ratio; TC—test centre.

^a^ 2 (paired/duplicated) samples excluded from accuracy analysis.

^b^ http://araw.mede.uic.edu/cgi‐bin/testcalc.pl.

**FIGURE 3:**
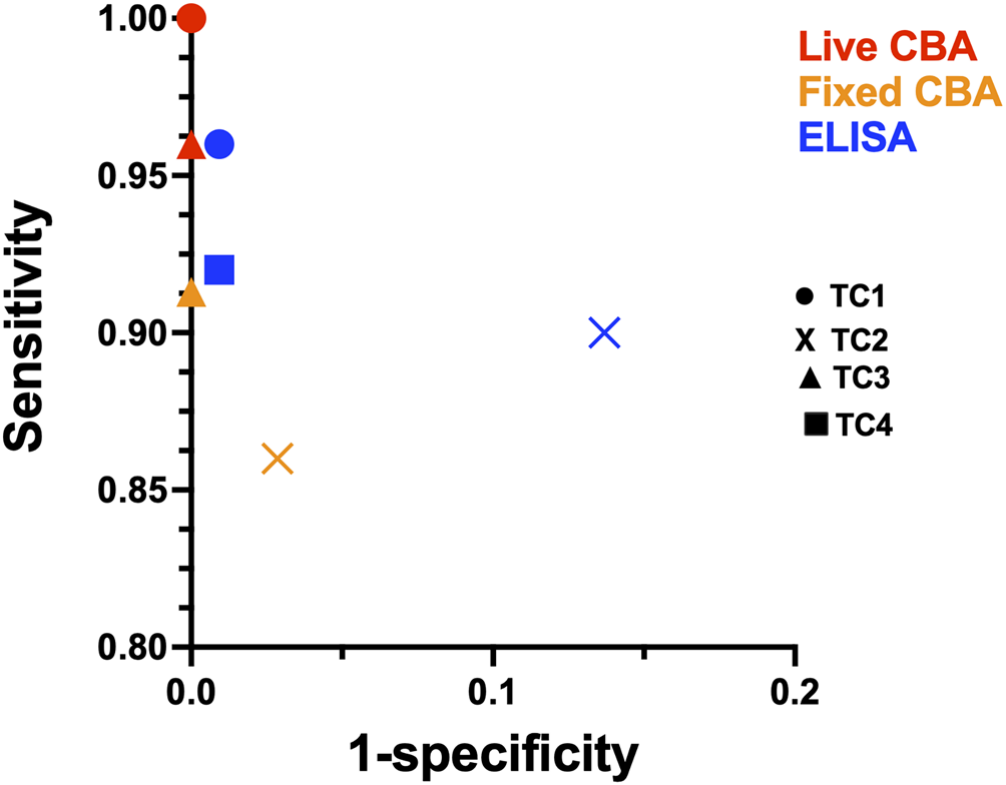
Sensitivity and specificity by assay type and testing centre. CBA—cell‐based assay; ELISA—enzyme linked immunosorbent assay.

Accuracy also varied between centres when broken down by assay type; live CBA (99.7%–100%), fixed CBA (96%–99%) and ELISA (87%–99%) (Figure 3). A teased nerve fibre assay was performed in one centre (TC2), and only on a limited number of ‘difficult’ or equivocal samples. Even allowing for this, this assay was positive in only 7/17 group 1 autoimmune nodopathy samples tested (37% sensitive), with 1/12 disease control samples also positive (83% specific). Overall, secondary assays, undertaken by 2/4 centres (TC1/2) had only a marginal and statistically non‐significant (*p* = 0.5, McNemar) effect on overall accuracy. In both cases, the combined approach was less sensitive but no more specific than the single best assay within the individual testing centre (Table A9).

### 3.4 Individual Antigens

**FIGURE 4:**
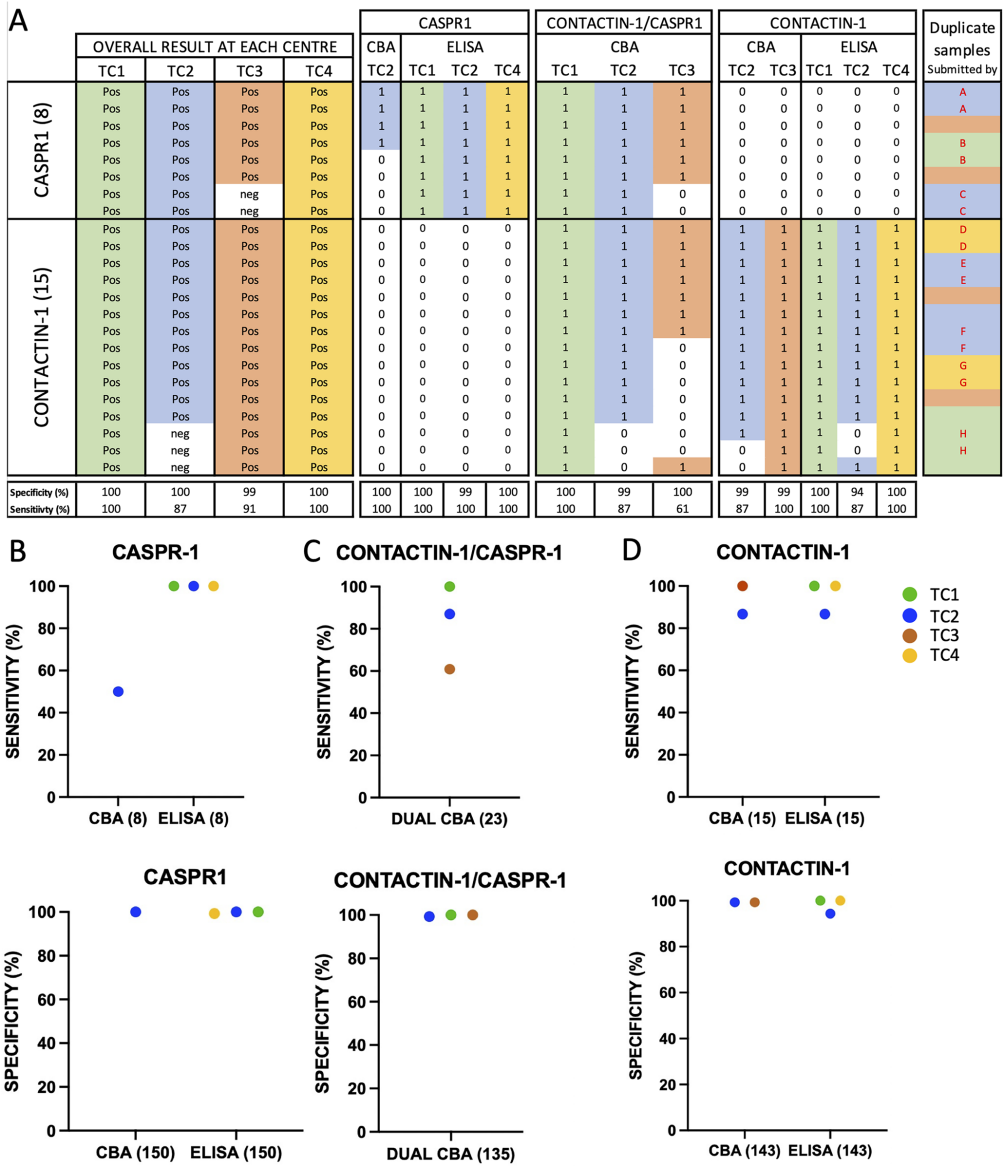
CASPR1/Contactin‐1 antibody testing. Samples positive for CASPR1 (8) and Contactin‐1 (15) were tested on 6 CBAs and 6 ELISAs at 4 centres (A). A positive result on any assay are coloured by testing centres (TC): TC1 (green), TC2 (blue), TC3 (brown) and TC4 (yellow). Negative results are shown in white. Test sensitivity and specificity for CASPR1 by CBA and ELISA (B), for Contactin‐1/CASPR1 dual CBA (C), and Contactin‐1 CBA and ELISAs (D) are shown above. These results are colour coded as in to (A). The colours in the right most column indicate the submitting lab, and the letters indicate duplicate samples. Caspr1—contactin associated protein 1; CBA—cell‐based assay; CNTN1—contactin‐1; ELISA—enzyme linked immunosorbent assay; TC—test centre.

**FIGURE 5:**
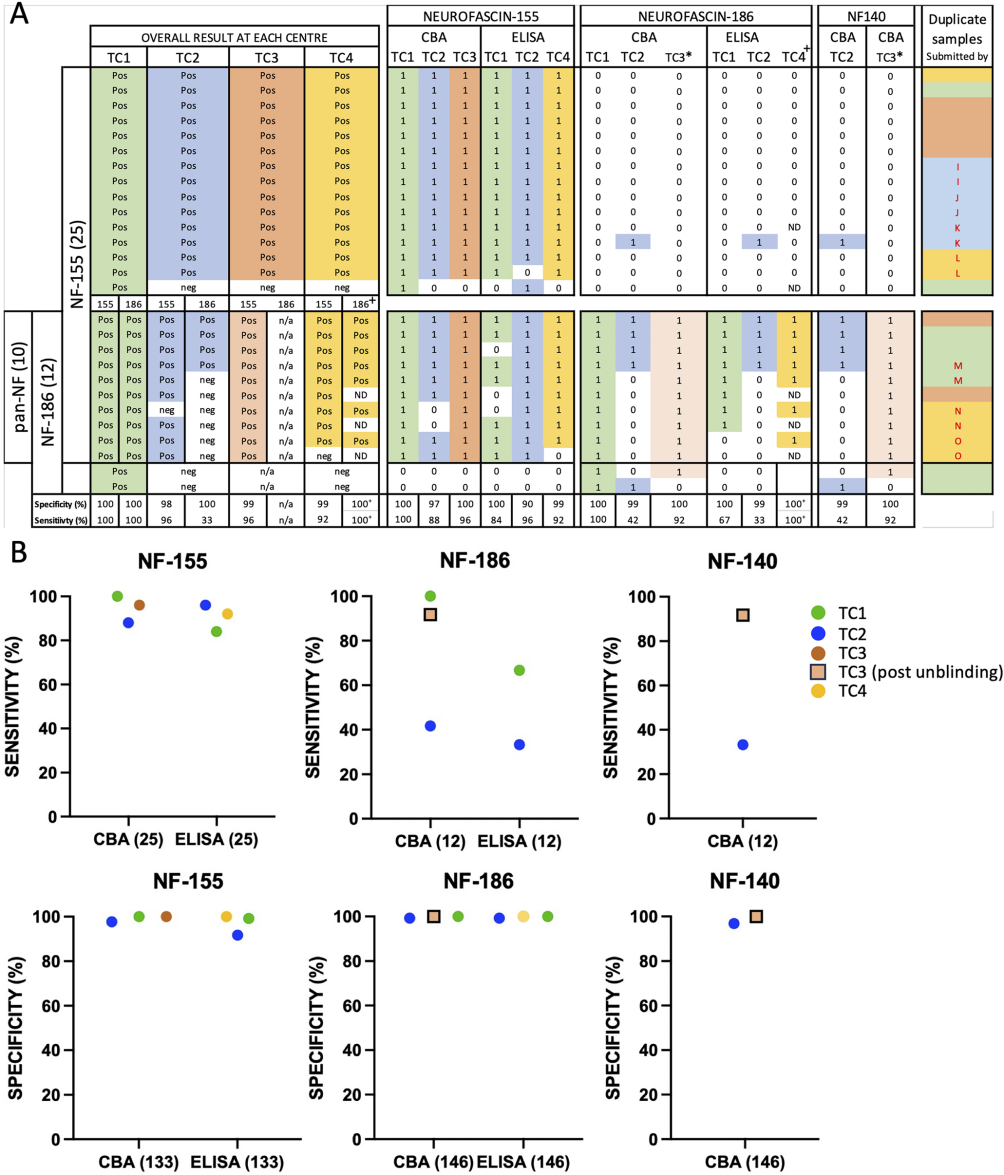
Neurofascin antibody testing. Samples positive for NF‐155 (15), NF‐186 (2) or both (10; labelled pan‐NF) were tested on 6 CBAs and 6 ELISAs at 4 centres. Additionally, a CBA for NF‐140 was run at two centres. Of note, assays for NF‐186 and NF‐140 at TC3 were run after the study was unblinded. (A) A positive result on any assay are coloured by testing centres (TC): TC1 (green), TC2 (blue), TC3 (brown), TC3 post unblinding (light brown) and TC4 (yellow). Negative results are shown in white. (B) Test sensitivity and specificity on an assay‐by‐assay basis, colour coded as before. * test performed after unblinding. + test not run on all samples. The colours in the right most column indicate the submitting lab, and the letters indicate duplicate samples. CBA—cell‐based assay; ELISA—enzyme linked immunosorbent assay; NF—neurofascin; TC—test centre.

To evaluate individual assays, their contribution to overall test performance, and to identify potential issues with specific assays or antigens, the raw data from each individual assay for each antibody target was then considered separately (Figures 4 and 5; Figure S1 and Table A5). In summary, this identified issues with the sensitivity of some assays, notably the fixed CNTN1 CBA in TC2 (87%), the CNTN1/Caspr1 dual CBAs performed by TC2 (87%) and TC3 (61%), the CNTN1 ELISA in TC2 (87%) and the NF155 ELISA in TC1 (84%). Similarly, for NF186, the sensitivities of the TC2 fixed CBA (42%, *p* = 0.0078), TC2 ELISA (33%, *p* = 0.0039), and TC1 ELISA (67%, ns, all McNemar) were all lower than that of the TC1 live CBA (100%). NF140 assays produced identical results to NF186 assays within, but not between, the different testing centres. In contrast, 15/26 assays were 100% specific, and 23/26 at least 99% specific, with, aside from the teased‐nerve fibre assay, the TC2 CNTN1 and NF155 ELISAs the least specific tests overall (94% and 92%, respectively).

### 3.5 End‐Point Titre

Relative end‐point titres were highly correlated between centres for Caspr1 (mean Spearman rank correlation coefficient (*r*_s_) = 0.88, range 0.86 to 0.90), but less well correlated for CNTN1 (*r*_s_ = 0.75, 0.69 to 0.79) and NF155 (*r*_s_ = 0.69, 0.62 to 0.76) (Figure S2).

### 3.6 Intra‐Assay Consistency

Short‐term, intra‐assay consistency was assessed using the 16 duplicated samples for Caspr1 (3), contactin‐1 (5), NF186 (1), NF155 (4), or both (panNF, 3). The same overall result was returned for all 16 by TC1 and TC2, and for 15/16 by TC3 and TC4. One aliquot of a duplicated sample, submitted as group 1 NF186 positive (at a borderline positive titre of 1:100) by TC2, was reported (NF155) positive by TC3 only. The other aliquot was reported as negative by all centres. A single other discrepant negative result was returned by TC4 for duplicate samples both judged to be NF155/panNF positive (at a titre of 1:800 to 1:900) by all other centres.

Analysing the 16 duplicated samples at the level of the individual assays, there were no significant differences in reproducibility between centres (TC1 111/112 [99%], TC2 135/144 [94%], TC3 47/48 [98%], TC4 45/46 [98%]). The different denominators here reflect the fact that different numbers and combinations of tests were performed for each sample across the different centres. Paired end‐point titres were identical for 39% of duplicated samples on average across all centres (range 33% to 42%), within 1 dilution step for 77.8% of samples, (Table A6), and varied by a median of 1 dilution step.

### 3.7 Inter‐Assay Consistency

Inter‐assay consistency was evaluated by comparing the results reported prior to submission of samples from a single testing centre with the blind repeat test results reported by the same testing centre. Two testing centres (TC1 and TC3) returned consistent overall results for all retested samples (including duplicates, both 100%, [41/41] and [40/40], whereas TC2 was consistent for 43/45 [96%] and TC4 for 32/34 [94%]). Three of the four inconsistent samples were positive only at the cut‐off titre (1:100) on one occasion and negative on every other test run across all centres. These samples were initially collected and tested between March 2018 and March 2019, whereas all 44 samples collected earlier than this returned consistent results on retesting by the same lab. As such, there was no evidence of a systematic effect of sample age on inter‐assay reproducibility.

The median inter‐assay variation in end‐point titre was also 1 dilution step, and was not significantly different (*p* = 0.44, Mann–Whitney) to that seen in the intra‐assay comparison. Paired end‐point titres were identical for 31.7% of repeats, and within 1 dilution step for 74.1% (Table A7).

### 3.8 Analysis of Discrepant Results

Discrepant results could be categorised into one of three groups (A–C), as below.

- Lower titre CNTN1/Caspr1 samples

TC2 failed to pick up 4/7 CNTN1 antibody positive samples with titres from 1:200 to 1:1000, but detected these antibodies in all 10 samples judged to have CNTN1 antibody titres above 1:1000 (according to the results of live CBA from TC1 or ELISA from TC4). Similarly, the TC3 dual CNTN1/Caspr1 CBA missed 9/23 positives, largely confined to lower titre CNTN1 (7/15) or Caspr1 sera (2/8), though all of the CNTN1 samples were ultimately correctly classified based in the results of the CNTN1 CBA. Furthermore, when the dual assay was repeated after unblinding with fresh, lower passage‐number HEK cells, background was reduced, and all previously false negative results were overturned.

B Low titre NF155 samples

Four of the nine discrepant NF155 results, including 2/3 of the duplicated pairs with discrepant results, involved samples with end‐point titres on the positive/negative threshold (1:100). Overall, the titres of the discrepant NF155 samples were 6.7‐fold lower than those which produced concordant results. Excluding panNF positive sera, TC2 returned 3 additional NF155 positive results compared to the other centres. Although two of these samples came from patients with a diagnosis of CIDP (with some features suggestive of autoimmune nodopathy, namely subacute onset and ataxia in both, tremor, pain and cranial nerve palsy in one each, with a poor response to IVIg and sustained improvement following corticosteroids), one came from a healthy control. In all three cases titres were either the lowest or second lowest reported for this antigen by this centre. Furthermore, none of these positive results were reproduced when fresh aliquots of the same sera were tested following unblinding. Classifying these results as false positive therefore seems justified.

C NF186 monospecific antibodies

Only 2 samples were reported as being positive for NF186 monospecific antibodies, by one centre (TC1), based on one assay (live CBA, at end‐point titres of 1:800 and 1:3200) and were negative by ELISA across all centres. Each sample was considered positive by CBA at one other centre. Both samples came from patients with immunotherapy responsive, CIDP‐like, inflammatory neuropathies. Again, both of these patients had clinical features in keeping with, but not specific for, autoimmune nodopathy; acute/subacute onset in both, and ataxia, pain or cranial nerve palsy in one each. One responded well but transiently to IVIg, with a more sustained response to corticosteroids, the other had no response to IVIg, a partial response to steroids, and a sustained response to rituximab.

### 3.9 Non‐Blinded Testing of Discrepant Samples

In order to evaluate the possible reasons for discrepant results, additional follow up testing was performed on a limited number of samples.

Three samples classified as CNTN1 positive by all labs except TC2 on blinded testing were retested on live and fixed CBA by TC2, and all three were positive on both assays, despite no known changes being made to this assay between runs. One of these samples was found to be positive on fixed CBA, but negative on ELISA and IHC, and one positive on ELISA, but negative on fixed CBA and IHC, during the blinded testing period. The sample from a patient with CIDP initially reported CNTN1 positive by TC3 only was considered negative upon later, unblinded retesting by the same lab. A new aliquot of the duplicate samples which were reported positive for Caspr1 by TC1, 2 and 4, but negative by TC3, was subsequently tested and also found Caspr1 positive by TC3, but with a very high background, providing a possible explanation for the initial false‐negative result.

Both of the group 2 samples found to be NF155 positive by TC2 only during the blinded testing period were found to be negative on subsequent follow‐up testing of new aliquots by the same lab, despite the original aliquots all remaining positive when retested in the unblinded period on both live and fixed CBA. The reasons for these discrepancies are unclear and a sample handing error or cross contamination cannot be excluded.

One of the two samples found to have monospecific NF186 antibodies by TC1 by live CBA, was subsequently also judged to be NF186 positive on live CBAs later performed by TC2 and TC3. The other sample was also found positive for NF186 on a live CBA by TC2 but not TC3. Both were negative when tested unblinded on the TC4 ELISA.

### 3.10 IgG Subclasses

Subclasses were determined at TC1, 2 and 4 on samples identified as seropositive. The cell‐based subclass detection system was more sensitive than ELISA, identifying 73 IgG subclass positive results across the four subclasses (21 IgG1, 18 IgG2, 13 IgG3 and 21 IgG4) from 44 sera tested, compared to a total of 45 from 31 and 38 from 44 for the two ELISAs. No subclass was detected, when all were tested for, in 2/42 (TC1), 0/31 (TC2) and 6/41 (TC4) samples. IgG4 was the most commonly detected subclass across all 3 centres, with the frequency of detection varying between 61% (27/44, TC1) and 97% (30/31, TC2) (Figure S3). Overall, there was a non‐significantly higher level of concordance for the dominant IgG subclass than for the presence or absence of individual subclasses (simple matching co‐efficient 0.74 vs. 0.71, *p* = 0.84, two‐tailed *T*‐test) (Table 3). Furthermore, there was on average greater concordance between the two ELISAs than between the ELISAs and CBA (0.89 vs. 0.64, *p* = 0.002, two‐tailed *T*‐test).

**FIGURE 6:**
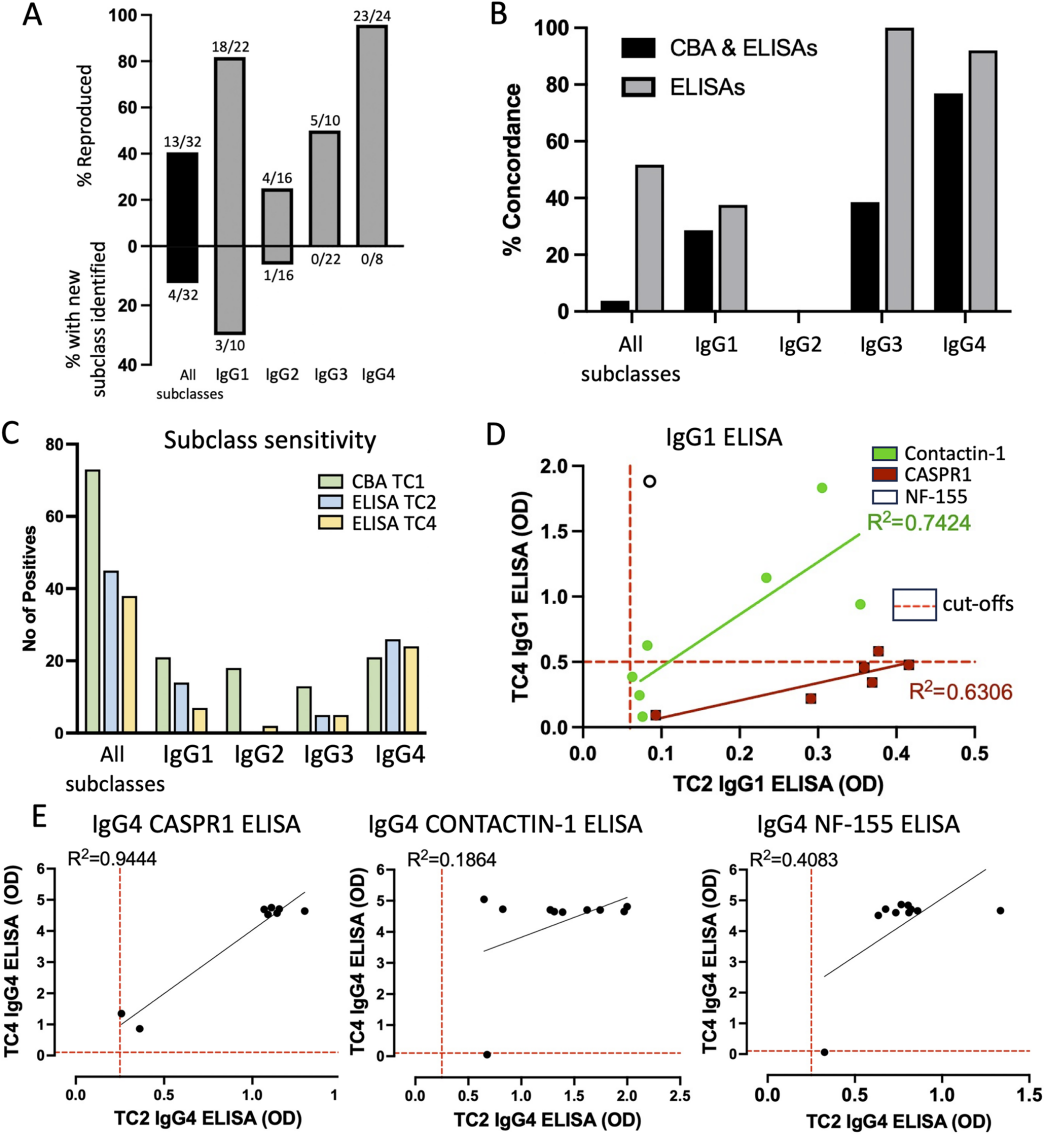
IgG subclasses. (A) Subclass reproducibility between pre‐submission results and blinded retesting. (B) Concordance between sub‐classes detected by all three assays (1 CBA and 2 ELISAs) or between the 2 ELISAs only. (C) Comparison of subclass detection rates between the different assays. (D) Although the raw data from the IgG1 ELISAs against CNTN1 (green) and Caspr1 (red) at TC2 and TC4 correlates well overall results show relatively poor concordance, due to differences in the OD threshold/cut‐off used to define positivity (red dashes). (E) There is greater variation in the correlations for IgG4 for the three antigens which appears to be driven by signal saturation within the TC4 ELISA. Caspr1—contactin associated protein 1; CBA—cell‐based assay; CNTN1—contactin‐1; ELISA—enzyme linked immunosorbent assay; NF—neurofascin; TC—test centre.

The subclass profile concordance (i.e., presence or absence of each of the four subclasses in an individual serum) between all centres was however poor, with only 1/26 samples tested at all three sites in complete agreement. Even between the two ELISAs concordance was poor for IgG1 (38%) and IgG2 (0%) (Figure 6). Although 5/6 samples for Caspr1 at TC4 were deemed IgG1 negative, there was a strong correlation in between the IgG1 ELISA scores at TC2 and TC4 across the six samples submitted as seropositive (*p* = 0.027, *R* ^2^ = 0.74; Figure 6). Differences in the ELISA cut‐off between the two centres appear to largely explain the discrepant results.

There was excellent concordance between the two ELISAs for the presence or absence of IgG4 antibodies with 24/26 positive on both assays. However, saturation in the IgG4 ELISAs, particularly at TC4, likely impacts on the correlation between the two centres for these assays (Figure 6).

### 3.11 Inter‐Assay Subclass Reproducibility

The original subclass data submitted was then compared with the blinded test results from the same centre. Only 13/32 (40%) serum subclass profiles were reproduced on blinded testing: IgG4 (23/24, 96%) and IgG1 (18/22, 82%) were the most reproducible individual subclass assays while IgG2 (4/16, 25%) and IgG3 (5/10, 50%) were relatively poor (Figure 6). Identification of the dominant IgG subclass was more consistent on blinded retesting, with an identical result reported for 23/24 (96%) of samples from the two centres where this data was available.

## 4 Discussion

This study shows that cell‐based assays (CBAs, mean accuracy 99%, range 97%–100%) and ELISA (97%, 91%–99%) are, in general, accurate and reliable methods to determine the presence or absence of nodal/paranodal antibodies. Other assays should be evaluated against these standards before being used in clinical practice. This applies equally to flow cytometry, western blot, i‐dots and recently developed commercial kits, all of which were not assessed by this study. Although the teased nerve fibre binding assay was only performed on a small number of ‘difficult’ samples in this study, the best‐case scenario for this particular assay (assuming correct categorisation of all remaining non‐tested samples) is an accuracy of 98% (sensitivity 83%, specificity 99%). However, given this assay was only performed in one centre, the generalisability of this observation to other centres and other teased nerve fibre assays cannot be confidently evaluated.

Tests for rare antibodies are often developed in‐house at individual centres as they are not attractive to commercial entities. Both ELISA and CBAs can perform inconsistently, and limited sample numbers can make it difficult to generate validation data. However, processes to maintain consistency of every aspect of the test should help reliability. This includes accurate monitoring of incubators, fridges and freezers, regular maintenance of microscopes and pipettes, careful culturing of cells and limiting their passage number. Developing monoclonal antibodies, specific for native proteins, as controls would also likely help with standardisation within and between laboratories. These checks should also help limit degradation of the assay performance over time.

In this study, the live CBA used by TC1 produced the highest accuracy. However, other, individual CBAs had reduced sensitivity (range 42%–100%) or specificity (97%–100%). The performance of ELISAs likewise varied, with a high rate of false positives (5/57, 9%) in one centre (TC2), yet high specificity (100%) in two others (TC1/4). Some authors attest that the clinical associations are more robust when only considering samples positive on ≥ 2 tests as ‘true positives’, and this approach is recommended by EAN/PNS CIDP guidelines [12]. However, in this study, the use of multiple assays had only a minimal, deleterious effect on accuracy, at best, and no combination improved on the specificity or accuracy of the single best assay within a given centre (Table A9).

Previous studies have suggested that the presence or absence of certain IgG subclasses, notably IgG4, is useful in the identification of a more specific clinical phenotype and different treatment response [14], whereas others have shown associations with IgG1, 2 or 3 [5, 15, 16]. The results of this study show that subclass detection is inconsistent between labs, especially for non‐IgG4 isotypes, with slightly higher levels of agreement on the ‘dominant’ IgG subclass. Notably, the two centres which used ELISA had generally higher levels of agreement with each other than with the one centre using live CBA for subclass determination. This does not appear solely due to generally increased sensitivity, as although the TC1 live CBA had higher rates of detection of IgG1‐3, IgG4 was detected less frequently. Some of the difference may also be due to variations in the subclass specific antibodies used by the different centres (Table A8). However, subclass profiles were also poorly reproducible within labs, suggesting these assays require further optimisation and better standardisation.

Further analysis of the discrepant results revealed useful information for assay development and quality control. The failure by TC2 to report sera with lower titre CNTN1 antibodies as positive suggested a problem with the sensitivity of these assays within this centre. On repeat unblinded testing of the 3 samples in question using both a live and fixed CBA, 2 were positive on both assays and 1 was positive in the live test only. Regular evaluation of low titre positive control samples on each assay should identify this type of issue early and allow remedial steps to be taken and the assay re‐run before results are finalised.

The relatively frequent discrepancies with NF155 antibody results, in particular those close to the positive/negative boundary, coupled with the observation that the NF155 assay producing the highest number of positive results was also the least specific, suggest that particular care should be taken in defining the positive/negative cut‐off and specificity on an assay‐by‐assay basis. Furthermore, there may be justification for defining an equivocal titre range where clinical significance is less certain.

Truly monospecific NF186 antibodies were *initially* only reported by one lab. Follow up testing suggests that these antibodies are likely to be best detected by live CBA, as both were *ultimately* deemed positive on at least 2 of 3 live CBAs and negative on 3 ELISAs (TC1, 2 and 4). Further studies are required to understand the reasons for this and the clinical and pathological implications of these results.

Similar to our findings, a previous study looking at ELISA for the detection of anti‐ganglioside antibodies also found a high level of agreement in positive/negative results overall, but systematic between‐laboratory differences in titres that were partially reduced by the use of standardised rather than in‐house methods [8]. Other multicentre studies have shown higher accuracy for live versus fixed cell‐based assays in the detection of MOG antibodies [17], and a higher sensitivity for live CBA than tissue‐based immunofluorescence in the detection of aquaporin‐4 antibodies in patients with neuromyelitis optica [18, 19]. In contrast, a recent single centre study found ELISA more sensitive than CBA for the detection of argonaute antibodies [11].

A major limitation of our study is the lack of antibody‐independent criteria for the diagnosis of autoimmune nodopathies. This means that assays were compared against each other, rather than to an underlying ‘gold standard’. The inclusion of samples from labs with higher sensitivity, or based on prior positivity (or negativity) on a single type of assay only, risks bias in favour of a particular lab or assay. Overall, the samples chosen for inclusion may have produced a small bias in favour of CBA and/or TC1. Prior to the study, 31 of the group 1 samples had been tested on both CBA and ELISA, and 3 of these were uniquely positive by CBA (at TC1), whereas 10 had only been tested on CBA and 11 only by ELISA. Similarly, 31 of the negative controls had not previously been tested by CBA, whereas 46 had not previously been tested by ELISA. However, on blinded retesting, 15 false positive results were produced by ELISAs and 4 by CBAs. Given these limitations, we do not recommend the use of one assay type over another, but instead suggest that the performance of any assay should be carefully evaluated and monitored.

In summary, for the routine, diagnostic testing of nodal/paranodal antibodies, we would recommend:

1. The use of an established and validated method, which could be either live CBA, fixed CBA or ELISA, depending on local experience and pragmatic considerations. Detailed protocols for each of the recommended techniques are provided in the Appendix S1.
2. The inclusion of low titre positive controls with each assay, run to monitor for loss of sensitivity over time, and regular sharing of low, high titre and negative controls between laboratories, on an annual or biannual basis.
3. Careful evaluation of the specificity of any newly established assay, using a large number of negative controls, and use of an equivocal titre range to indicate results with less certain clinical significance.
4. Caution in attaching significance to small or even moderate changes (≤ 2 dilution steps) changes in end‐point titre, especially if both samples are not assayed within a single test run.
5. A standardised terminology for describing antibody specificity (Table 4).

**TABLE 3:** IgG subclass testing concordance.

		CBA versus ELISA		ELISA versus ELISA	Mean
		TC1 versus TC2	TC1 versus TC4	TC2 versus TC4	
Present	IgG1	0.75	0.50	0.68	0.64
	IgG2	0.43	0.50	0.93	0.62
	IgG3	0.68	0.68	1.00	0.79
	IgG4	0.75	0.75	0.93	0.81
	Mean	0.65	0.61	0.88	0.71
Dominant		0.72	0.61	0.90	0.74

*Note:* Simple matching co‐efficient for the presence or dominance of IgG subclasses.

Abbreviations: Caspr1—contactin associated protein 1; CBA—cell‐based assay; CNTN1—contactin‐1; ELISA—enzyme linked immunosorbent assay; IHC—immunohistochemistry; NF—neurofascin.

**TABLE 4:** Recommended terminology for classifying antigenic target.

Results on individual assays					Recommended terminology
NF155	NF186	CNTN1/Caspr	CNTN1	Caspr1	
+	−	−	−	−	NF155 monospecific
−	+	−	−	−	NF186 monospecific
+	+	−	−	−	Pan‐neurofascin
−	−	+	+	−	Contactin‐1
−	−	+	−	+	Caspr1
−	−	+	−	−	CNTN1/Caspr1 complex^a^
−	−	+	+	+	CNTN1 and Caspr1

*Note:* In this study all NF140 positives were also positive against NF186. As such, a sperate category for NF140 is not included.

Abbreviations: Caspr1—contactin associated protein 1; CNTN1—contactin‐1; CNTN1/Caspr1—indicates co‐transfection for CBA; NF—neurofascin.

^a^ There is some evidence to suggest that this pattern is consistent with reactivity against Caspr1 alone.

We would also suggest that further work is needed to harmonise IgG subclass detection and reporting (likely through the use of standardised methods, reagents and thresholds/definitions of positivity), and to evaluate the clinical and pathological relevance of NF186 monospecific antibodies and the different IgG subclasses to autoimmune nodopathies.

## Conflicts of Interest

S.R. is a Medical Advisory Board member of the Guillain‐Barré syndrome and Associated Inflammatory Neuropathies (GAIN) patient charity. His department receives payment for the provision of diagnostic antibody testing, including of nodal/paranodal antibodies. He has received payment from Argenx for preparation and delivery of conference presentation on clinical trial, payment for serving on argenx, Annexon, Dianthus and Hansa clinical trial advisory boards, and payment from CSL for delivering a conference symposium lecture. Kathrin Doppler has received honoraria for lectures and presentations from Takeda, Grifols, Sanofi and Roche, and is a board member of the Inflammatory Neuropathy Consortium of the Peripheral Nerve Society. L.A. and K.D. have been supported by a scholarship from the Interdisciplinary Center of Clinical Research (IZKF) of the Faculty of Medicine, University of Würzburg (ZZ‐32, AdvCSP‐1) unrelated to the contents of this manuscript. R.H. is editorial board member of the Journal of the Peripheral Nervous System, a board member of the Inflammatory Neuropathy Consortium of the Peripheral Nerve Society, and reports funding from BÜHLMANN Laboratories AG outside the submitted work. Erasmus MC offers diagnostic services for testing of paranodal antibodies. M.T. was supported by the Erasmus MC Pain Foundation, has received funding from ZonMw (Memorabel programme), the Dutch EpilepsieNL Foundation (NEF 19‐08), Dioraphte (2001 0403) and E‐RARE JTC 2018 (UltraAIE, 90030376505). M.T. has filed a patent, on behalf of the Erasmus MC, for methods for typing neurological disorders and cancer, and devices for use therein, and has received research funds for serving on a scientific advisory board of Horizon Therapeutics/AmGen and ArgenX, for consultation at Guidepoint Global LLC, for consultation at UCB. MT has received an unrestricted research grant from CSL Behring. M.T. has received royalties from UpToDate Inc. L.Q. is supported by INT23/00066 and PI22/00387 from Instituto de Salud Carlos III—Ministry of Economy and Innovation (Spain) and received research grants from CIBERER, Fundació La Marató, GBS‐CIDP Foundation International, UCB and ArgenX. L.Q. received speaker or expert testimony honoraria from CSL Behring, Novartis, Sanofi‐Genzyme, Merck, Annexon, Alnylam, Janssen, ArgenX, UCB, Dianthus, LFB, Avilar Therapeutics, Lycia Therapeutics, Nuvig Therapeutics, Takeda and Roche. L.Q. serves at Clinical Trial Steering Committees for Sanofi Genzyme and Takeda is Principal Investigator for UCB's CIDP01 trial and Sanofi's Mobilize and Vitalize trials. C.S. is supported by Deutsche Forschungsgemeinschaft (SFB1158, CRU5001 and RTG2026), unrelated to the content of this manuscript, and she is a Board Member of the Peripheral Nerve Society. She has served as a scientific advisor for Algiax, Grifols, Immunic, Kedrion, Sanofi, and Takeda, and has given educational talks for Alnylam, Argenx, CSL Behring, Grifols, Kedrion, and Takeda. P.W. is a named inventor on patents for antibody assays and has received royalties. He has received honoraria from Biogen Idec, Mereo Biopharma, Retrogenix, UBC, Euroimmun AG, UCB, F. Hoffmann La‐Roche and Alexion; travel grants from the Guthy‐Jackson Charitable Foundation; and research funding from Euroimmun AG. Work in the Autoimmune Neurology Diagnostic Laboratory is supported by the NHS Commissioning service for NMOSD. Hugh Willison, Susan Halstead, Victor Mgbachi, and Sophia Rohrbacher report no conflicts of interest.

## Supporting information


**Appendix S1.** Supporting Information.

**Figures S1–S3.** Supporting Information Figures S1–S3.

**Tables A1–A9.** Supporting Information Tables A1–A9.

## Data Availability

The data that support the findings of this study are available from the corresponding author upon reasonable request.
