# Supplementary material for: Inter‐Laboratory Validation of Nodal/Paranodal Antibody Testing
Source: J Peripher Nerv Syst. 2025 Jan 29;30(1):e70000. doi: 10.1111/jns.70000 (PMC11780190; doi:10.1111/jns.70000)
Supplement: Supplementary file 1 — Appendix S1. Supporting Information. [file JNS-30-0-s001.docx]

**Appendices**

**1. Protocol for inter-laboratory validation of nodal/paranodal antibody testing**

# Testing laboratories

In alphabetical order (not TC number order):

Barcelona (Querol)

Oxford (Rinaldi)

Rotterdam (Huizinga/Titulaer)

Würzburg (Sommer/Doppler)

# Co-ordinating, blinding and sample distribution centre

- Glasgow (Willison)

# Rationale

To assess the inter-laboratory variability in nodal/paranodal testing and to establish reference laboratories and testing standards to facilitate the harmonisation and consistency of further test centres

# Protocol

Each lab is to identify, from its own test cohort, 25-40 serum samples that they consider to be:

1. Positive for any nodal/paranodal antigen, and clearly from a patient with the relevant clinical phenotype (i.e. an immunotherapy responsive “inflammatory” / autoimmune peripheral neuropathy) (~5-10 samples)
2. Negatives from patients with confirmed inflammatory neuropathies (~10-15 samples)
   1. Borderline/equivocal/’difficult’ sera (but nevertheless considered negative and from a definitely clinically irrelevant population, e.g. MGUS without neuropathy) (~1-5 samples) and
   2. negatives from other unrelated diseases or healthy or healthy controls (~10-15 samples)

For each sample, at least 300 ml (and ideally 600 ul where available) serum is to be sent to the co-ordinating centre. If samples need to be defrosted to aliquot them prior to sending, they should not be refrozen but instead sent defrosted on wet ice.

Testing labs will co-ordinate such that they are able to send their samples to the co-ordinating lab on the same day. They will also simultaneously provide to the co-ordinating lab their own laboratories’ results for each sample submitted (dichotomised to positive or negative) and basic demographic and clinical details to include:

- Age / sex
- Diagnosis / diagnostic category (1-3a/3b, as above)

For positive samples, additional detail should be provided on:

- The specific target antigen(s) (NF155, NF140/186, CNTN1, CNTN1/Caspr1, Caspr1)
- The end point titre and the method used to titrate the sample.
- Subclass (positive/negative for each, with predominant subclass, or end-point titre for each subclass, as per local laboratories usual practice)

A template will be created and used by all centres to record and submit these details. Each centre will also provide a copy of their testing protocol to the co-ordinating centre at the time the samples are submitted.

This information will be kept confidentially by the co-ordinating centre until all repeat assay results have been submitted.

The co-ordinating centre will then, within 2 weeks of receiving all samples:

1. Recode each sample
2. Produce 5 identical 60 μL aliquots from each sample
3. Double code and aliquot a proportion of the positive and negative samples, where 600 ul is available, at their discretion
4. Distribute 1x 60 μl of each coded sample (about 140 in total) to each of the testing labs (on ice), reserving 1 x 60 μl +/- 1 x 300 μl of each in the co-ordinating centre, refrozen and stored at -80°C.

Samples should only be defrosted once between being sent in by the testing labs and back out by the co-ordinating lab.

The testing centres will then test each recoded sample in a blinded fashion, returning their results to the co-ordinating centre as before, within 12 weeks of receipt of the samples.

On receipt of the recoded samples, the testing labs should store these as per their standard operating procedure (which should be recorded), until their own testing is complete, with any residual sample then frozen at -80°C.

Each centre will perform testing as per their usual protocol. If a centre usually uses multiple assays (eg. CBA, ELISA, teased nerve) then the results on each assay should be returned, along with the overall classification of each sample as positive or negative. If subclass testing and end-point titration are performed routinely by each lab, these results should also be returned. A standardised template for returning assay results will be used.

When all results are received by the co-ordinating centre, the co-ordinating centre will collate the results. Any “positive” sample submitted by a lab which is reported as negative on blinded retesting by the same lab (and all other labs) will be excluded from the inter-lab comparison. The co-ordinating centre will then produce a blinded summary comparing the 4 testing labs’ results in each of the 3 original sample categories, presented as follows:

| Group and original result | Percentage returned as positive on blinded testing | | | |
| --- | --- | --- | --- | --- |
|  | Lab 1 | Lab 2 | Lab 3 | Lab 4 |
| 1. Positive (inflammatory neuropathy) |  |  |  |  |
| 2. Negative (inflammatory neuropathy) |  |  |  |  |
| 3. Negative (healthy and clinically irrelevant controls) |  |  |  |  |

For blindly pre-titrated sera, only the results from the initial, undiluted sample will be used in the above assessment.

The primary analysis will be based on the accuracy of each labs’ final results, assuming:

1. A background prevalence of positive results in the routinely tested population of 7.5%
2. All samples in group 1 are true positives, as long as detected by at least one lab, and even if not detected by any other labs
3. All samples in groups 2 and 3 are true negatives

Accuracy will then be calculated as:

0.075(sensitivity) + 0.925(specificity)

Additionally, the following comparisons will be made:

1. Analysis of different classes of assays (e.g. live CBA, fixed CBA, ELISA, Western blot)
2. Inter-laboratory variability assessment of subclass identification and titre
3. Intra-laboratory variability over time (each lab will have blindly retested its own samples after a delay)
4. Short term intra-laboratory variability (derived from double coded samples)

Secondary outcomes will be based on the consistency of the identified antigen, subclass and titre.

The results of this study will be reported in peer-reviewed journals to provide a recommended testing standard and inform future diagnostic guidelines.

Involved laboratories should then consider setting up an ongoing quality and consistency assurance programme, with low numbers of samples sent intermittently for testing in all participating labs.

**2. Supplementary methods**

**2.1 Recommended protocol for live CBA (from TC1)**

**2.1.1 Materials**DMEM only

DMEM with 10% FCS

DMEM/HEPES (2.3 g HEPES in 500 ml DMEM)

PBS

DMEM/HEPES/BSA (add 1% BSA to the DMEM/HEPES solution)

HEK293 cells

Poly-L-Lysine (prepare stock at 1 mg/ml – dilute to 20 mg/ml in PBS for coating)

Jet PEI

4% PFA

**Antibodies**

| **Antibody** | **Source** | **Cat no** |
| --- | --- | --- |
| Human/Mouse/Rat Neurofascin Antibody | R&D | AF3235 |
| Mouse anti-human IgG1 (unconjugated) | Sigma | I2513-.2ML |
| Mouse anti-human IgG2 (unconjugated) | Sigma | I5635-.2ML |
| Mouse anti-human IgG3 (unconjugated) | Sigma | I7260-.2ML |
| Mouse anti-human IgG4 (unconjugated) | Sigma | I7385-.2ML |
| Human Contactin-1 Affinity Purified Polyclonal Ab | R&D Systems | AF904-SP |
| Caspr1 | Neuromab | 75-001 |
| F(ab')2-Goat anti-Human IgG Fc Secondary Antibody, Alexa Fluor 488 | Thermo Fisher | h10120 |
| Goat anti-Mouse IgG (H+L) AF488 | Life Tech | A11029 |
| Goat anti-Chicken IgY (H+L) AF546 | Life Tech | A11040 |

**Plasmids**

| **Target** | **Vector** | **Sequence ref** | **Source** | **Cat no** |
| --- | --- | --- | --- | --- |
| **NF155** | pCMV6- NFASC-Myc-DDK | NM_001160331 | Origene | RC228652 |
| **NF186** | pcDNA3.1 –Nfasc186-Myc | NM_001005388 | Jerome Devaux, University of Marseille | n/a |
| **CNTN1** | pReceiver-MO2-CNTN1 | NM_001843.3 | GeneCopoeia | EX-A1153-M02 |
| **Caspr1** | pReceiver-MO2-CNTNAP1 | NM_003632.2 | GeneCopoeia | EX-M0417-M02 |

**2.1.2 Method**

1. Day 1 – place air dried, acid-treated, ethanol washed, 13mm coverslips into a 24 well plate and coat coverslips only with 70 ml 20 mg/ml PLL. Leave for 15 min. Aspirate and leave to air dry.
2. Lift stock HEK293 cells with TrypLe, spin down 500 g / 5 mins, resuspend in 1 ml DMEM with 10% FCS. Count.
3. Plate 5x10^4^ cells/well in total 70 ml media / well (DMEM, 10% FCS). Top up to 250 ml by end of day and leave to grow o/n in 37^o^C incubator with 5% CO_2_.
4. Day 2 – When cells reach 60%-80% confluence transfect with DNA of choice.
   1. 1 mg DNA + 2 ml JetPEI / well (NF155, NF186, or NF140)
   2. 0.5 mg CNTN1 DNA, 0.5 mg Caspr1 DNA + 3 ml JetPEI (for CNTN1/Caspr1 co-transfection)
   3. 1 ug DNA + 3 ml JetPEI (CNTN1 or Caspr1 if singly transfected)
   4. For each serum to be tested, also include a mock-transfected control well (2 ml JetPEI only)
      - Prepare DNA and PEI separately in 150 mM NaCl to 50 ml/well total volume each. Vortex and spin down briefly.
      - Add PEI to DNA dropwise. Vortex and spin down briefly.
      - Incubate 15 minutes room temperature (RT)
      - Add 100 ml complex mix / well dropwise (distributing around coverslip).
      - Homogenise by gentle swirling.
      - Incubate overnight (NF155, 186, 140) or 6 h (CNTN1, Caspr1 – CNTN1 especially appears toxic to the cells with prolonged transfection times).
5. Wash cells with 250 ml medium/well and add 250 ml fresh medium.
6. Day 4 (at least 24 h after transfection) - Dilute serum 1:100 (NF155, NF186) in DMEM/HEPES/BSA with 1:1000 CK anti-NF or 1:40 (CNTN1, Caspr1) in DMEM/HEPES/BSA and add to the wells (250 ml) for 1 hr at RT.
7. Wash x 3 with DMEM-HEPES
8. Fix with 4% Formaldehyde in PBS for 5 min at RT.
9. Rinse in PBS x3
10. Incubate with secondaries for 45 min, RT.
    1. NF155/186: goat anti-human IgG Fc AF488 (1:750) and anti-CK AF546 (1:1000) in DMEM/HEPES/BSA
    2. CNTN1/Caspr1: goat anti-human IgG Fc AF488 (1:750) in DMEM/HEPES/BSA
11. Wash x3 DMEM/HEPES and x2 in PBS
12. Incubate with DAPI (1:50,000) for 5 minutes. Rinse 2x PBS.
13. Read assay in plates using fluorescent microscopy or alternatively/additionally mount on slides with mounting medium for imaging. Human IgG binding is scored on a 5 point scale taking into account fluorescence intensity and co-localisation with the commercial antibody (0 – negative, 1 – low positive, 2-4 positive with fluorescnce intensity below, equivalent or greater than that seen with the commercial).

**Notes:**

Non-specific binding can be a problem with the NF assays in particular. Try to avoid the cells being confluent or clumped when running the assays, as this seems to contribute to the problem and can also reduce the intensity of the commercial antibody co-stain.

When analysing these results pay attention to whether there is good co-localisation between the serum IgG signal (green) and the commercial pan-neurofascin antibody (red). For true positives there should be.

Patients with CNTN1 antibodies (almost) always show faint membrane staining on the mock-transfected HEKs, as these cells have low-level endogenous CNTN1 expression.

Be aware that some sera can react strongly against HEKs regardless of the transfection, and these rarely show CNTN1 specific binding. ELISA can help in all the above situations.

**For all positives**

1. Repeat the assay against the same antigen(s) and additionally **check titre** by serial doubling dilution from 1:100 (to 1:200, 1:400, 1:800. 1:1600, 1:3200, and 1:6400). The end-point titre is the highest titre scored as 1 or above.

2. **Identify the subclasses** present by replacing the goat anti-human IgG Fc AF488 secondary with the following subclass specific antibodies (all at 1:100 in DMEM/HEPES/BSA) in 4 separate wells (keep serum at 1:100 for NF and 1:40 for CNTN1/Caspr1):

| Mouse anti-human IgG1 (unconjugated) | I2513-.2ML |
| --- | --- |
| Mouse anti-human IgG2 (unconjugated) | I5635-.2ML |
| Mouse anti-human IgG3 (unconjugated) | I7260-.2ML |
| Mouse anti-human IgG4 (unconjugated) | I7385-.2ML |

Incubate for 45 min, RT.

Wash x3 DMEM/HEPES and x2 in PBS.

Incubate with tertiary - goat anti-mouse 488 (Life Tech A11029) 1:1000 in DMEM/HEPES/BSA, 45 min, RT.

Wash x3 DMEM/HEPES and x2 in PBS

Incubate with DAPI (1:50,000) for 5 minutes. Rinse 2x PBS.

Read assay as before.

**For positives on CNTN1/Caspr1 co-transfected cells**

1. Also repeat assay at original serum concentration against both CNTN1 and Caspr1 singly transfected cells (to establish if CNTN1+, Caspr1+ or CNTN1/Caspr1 complex+ only)

**If using Caspr1 commercial primary antibody** (for co-localisation), after PFA fixation and rinse, permeabilise with ice-cold methanol, 400 μl/well, for 30 mins on ice. Wash 4 x PBS.

Incubate with CASPR1 antibody at 1:1000 in DMEM/HEPES/1% BSA for 1 h at RT.

Wash 3 x DMEM/HEPES, 1 x PBS.

Include anti-mouse AF546 (1:1000 - for Caspr1 primary) in DMEM/HEPES/1% BSA in secondaries.

NB The Caspr1 commercial is not compatible with subclass testing. It is not recommended to use this commercial routinely.

**2.2 Recommended protocol for fixed CBA (from TC3)**

**2.2.1 Materials**

- 8-well chamberslides (Nunc Lab-Tek)
- Gelatin 2.5%
  - 100 ml MilliQ
  - 2.5 g Gelatin
  - Autoclave before use
- DMEM 10% heat-inactivated FBS 1% penicillin/streptomycin (DMEM++)
- DMEM only
- HEK293 cells
- Fugene HD transfection reagent (Promega)
- 4% PFA
  - 100 ml Distilled water (little bit pre-heated)
  - 4 g PFA
  - 0.4 g NaOH
  - Once dissolved add 1.6 g Sodium Phosphate Monobasic
- 0,2% Triton/PBS
  - 100 ml 1x PBS
  - 2 ml 10% Triton X-100
- 1% BSA/PBS
  - 100 ml 1x PBS pH 7.8
  - 1 g BSA
- Vectashield Hard-Set with DAPI (Vector laboratories – H1500)

**Antibodies**

| **Antibody** | **Source** | **Cat no** |
| --- | --- | --- |
| Goat Anti-human Contactin-1 Affinity Purified Polyclonal Ab | R&D Systems | AF904 |
| Rabbit Anti-human Caspr1 | Abcam | ab133634 |
| Rabbit Anti-Goat AF488 | ThermoFisher | A-11078 |
| Goat Anti-Rabbit AF488 | ThermoFisher | A-11008 |
| Donkey Anti-Human IgG Cy3 | Jackson Immuno Research | 709-166-149 |
| Mouse Anti-human IgG1-AF555 | Southern Biotech | 9052-32 |
| Mouse Anti-human IgG2-AF555 | Southern Biotech | 9070-32 |
| Mouse Anti-human IgG3-AF555 | Southern Biotech | 9010-32 |
| Mouse Anti-human IgG4-AF555 | Southern Biotech | 9200-32 |

**Plasmids**

| **Target** | **Vector** | **Sequence ref** | **Source** | **Cat no** |
| --- | --- | --- | --- | --- |
| **CNTN1** | pReceiver-M02-CNTN1 | NM_001843.3 | Genecopoeia | EX-A1153-M02 |
| **Caspr1** | pReceiver-M02-CNTNAP1 | NM_003632.2 | Genecopoeia | EX-M0417-M02 |

**2.2.2 Methods**

**Day 1 Seeding the cells**

1. Coat the 8-well LabTek with 300 μl/well Gelatin 2,5%
2. Incubate 30 min. at room temperature (RT; 21 °C)
3. Harvest HEK293 cells (25 cm^2^ flask; 80-90% confluent), wash and resuspend in 5 ml DMEM++
4. Take off the Gelatin from the LabTek wells
5. Dilute HEK293 cells 20x for transfection the next day (24 hrs.), or 40x for transfection the day after (48 hrs.). Seed 500 μl cell suspension per well.
6. Incubate 24-48 hrs. at 37°C with 5% CO_2_

**Day 2 Transfection**

1. Make a DNA/fugene mix (ratio 1:3)
   1. Add 20 μl DMEM only to a tube
   2. Add 1 μg DNA to the DMEM only (for CNTN1/CASPR1 use 0.5 μg of each plasmid)
   3. Mix and incubate 5 min. at RT
   4. Add 3 μl fugene to the DNA mix
   5. Mix and incubate 5-15 min. at RT
2. Add the mix to the well (24 μl/well)
3. Incubate 18 hrs. at 37°C with CO_2_

**Day 3 Fixation**

1. Take off the medium from your cells (try to work fast, cells are still alive)
2. Add 300 μl/well 4% PFA (to fix the cells)
3. Incubate 15 min. at RT
4. Take off the 4% PFA
5. If not proceeding immediately, add 300 μl PBS and store slides at 4 °C

**Day 3 Immunofluorescence**

1. Carefully remove the chambers from the slide. Put the slides into a coplin jar with PBS.
2. Wash 5 min. with 0.2% Triton/PBS at RT (to permeabilize the cells). Use shaker at 150 rpm.
3. Wash 3 times 5 min. with PBS at RT. Use shaker at 150 rpm.
4. Remove slides from coplin jar, dry edges and add 40 μl 1% BSA/PBS per well (for blocking)
5. Incubate 1hr. at RT in dark and moist chamber
6. While incubating dilute sera 1:100 in 1% BSA/PBS (for subclasses use 1:10)
7. Tap off 1% BSA/PBS from slide
8. Put on 40 μl Primary Antibody per well
9. Incubate 1 hr. at RT in dark and moist chamber
10. Wash 3 times 5 min. in a coplin jar with PBS at RT. Use shaker at 150 rpm.
11. While washing prepare Commercial Antibodies (1:100 CNTN1; 1:1000 Caspr1) in 1% BSA/PBS
12. Put on 40 μl Commercial Antibody per well
13. Incubate 1 hr. at RT in dark and moist chamber
14. Wash 3 times 5 min. in a coplin jar with PBS at RT. Use shaker at 150 rpm.
15. While washing prepare Secondary Antibody in 1% BSA/PBS (Donkey Anti-Human IgG CY3 1:200 and Rabbit Anti-Goat AF488 or Goat Anti-Rabbit AF488 1:1000; Mouse Anti-human IgG1-4 AF555 1:500)
16. Put on 40 μl Secondary Antibody per well
17. Incubate 1 hr. at RT in dark and moist chamber
18. Wash 3 times 5 min. in a coplin jar with PBS at RT. Use shaker at 150 rpm.
19. Cover with Vectashield (hard set with DAPI) and coverslip (15 μl Vectashield per well)
20. Store the slides at 4 °C

**2.2.3. Interpretation**

1. Negative and positive control sera should be included in every experiment.
2. Check staining for CNTN1 or CNTN1/Caspr1 as visualized by commercial antibodies using a fluorescence microscope (e.g. Leica DM-RXA, Meyer Instruments). Both transfected and non-transfected cells should be observed. The non-transfected cells are used as reference for background staining of patients’ IgG.
3. Assess co-localization of patients’ IgG with CNTN1 or Caspr1 positive cells. Only if human IgG staining co-localizes with CNTN1 or Caspr1 positive cells, it should be considered positive. Caveat is a non-specific staining of non-transfected cells (without co-localization). Review is typically performed by two independent researchers. In doubt, a third assessor should be asked to observe the slides, or repetition or additional tests should be performed.
4. Re-test all positive samples in a separate experiment for confirmation.

**2.2.4 Notes**

If cells detach during the staining procedure, washing steps can be performed without shaking.

**2.3 Recommended protocol for ELISA (from TC4)**

2.3.1 Materials

- Nunc-Immuno 96 Microwell^TM^ ELISA plates
- 0.1M PBS
- Blocking solution: 200ml 0.1 M PBS with 100μl 20 Tween und 6.66ml 10% BSA/PBS
- Washing buffer: 1 liter 0.1 M PBS with 500μl Tween20
- TMB Solution
- 1 M H_2_SO_4_

2.3.1.1 Proteins/antibodies

| Protein/antibody | source | Cat. No. |
| --- | --- | --- |
| Neurofascin-155 protein | In our assay: Kindly provided by E. Meinl^*^  Alternatively use: Origene | TP328652 |
| Neurofascin-186 protein | In our assay: Kindly provided by E. Meinl^*^  Alternatively use: Origene | TP329070 |
| Contactin1 protein | SinoBiological | 10383-H08H |
| Caspr1 protein | R&D Systems | 2418-CR |
| Pan-neurofascin antibody | R&D Systems | AF3235 |
| Contactin1 antibody | Abcam | ab191285 |
| Caspr1 antibody | Santa Cruz | Sc-373777 (E-8) |
| Anti-human IgG, HRP conjugated | Jackson Immuno Research | 109-035-006 |
| Anti-chicken IgG, HRP conjugated | Thermo Fisher Scientific | PA1-28798 |
| Anti-goat IgG, HRP conjugated | Abcam | ab205723 |
| Anti-mouse IgG, HRP conjugated | Jackson Immuno Research | 715-035-150 |
| Anti-human IgG1, HRP conjugated | Thermo Fisher Scientific | A-10648 |
| Anti-human IgG2, HRP conjugated | Thermo Fisher Scientific | 05-0520 |
| Anti-human IgG3, HRP conjugated | Thermo Fisher Scientific | 05-3620 |
| Anti-human IgG4, HRP conjugated | Thermo Fisher Scientific | A-10654 |

^*^ Ng et al. 2012, https://pubmed.ncbi.nlm.nih.gov/23100406/

2.3.2 Protocol

1. Coat Nunc-Immuno 96 Microwell^TM^ ELISA plates with 100 μl/well of NF155 (5μg/ml in PBS), NF186 (5μg/ml in PBS), CNTN1 (2μg/ml in PBS) or Caspr1 protein (2μg/ml in PBS) by incubation overnight at +4°C. Half of the wells need to remain uncoated as a control and are filled with PBS (100 μl/well).

2. Wash twice with 200 μl PBS, following tapping out of persisting fluids.

3. Incubate with 200μl blocking solution (0.1M PBS, 3.33% BSA, 0.05% TweenR 20) per well for 1h at 37°C on a shaker.

4. Wash twice with washing buffer (0.1M PBS, 0.05% TweenR20), following tapping out.

5. Dilute patient sera 1:100 with blocking solution; for control antibodies the following dilutions are used: anti-pan-neurofascin 1:1000, anti-contactin1 1:400, anti-caspr (1:5000).

6. Put 100 μl of diluted sera/control antibodies or negative controls (blocking solution) into the wells. All samples should be tested in duplicate. Each sample is tested on coated and uncoated wells in parallel.

7. Incubate the samples for one hour at 37°C on a shaker.

8. Wash each well four times following tapping out persistent fluids.

9. Add the following secondary antibodies (diluted in blocking solution): anti-human IgG 1:10000 for human samples, anti-chicken IgY 1:10000 for the anti-pan-neurofascin control, anti-goat IgG for the anti-contactin1 control, anti-mouse IgG 1:1000 for the caspr1 control. Incubate them for 30 min at 37°C on a shaker.

10. Wash each well six times, then add 100 μl of TMB solution per well and incubate for exactly 15 min in the dark.

11. Then add 50 μl of 1 M H_2_SO_4_ per well and measure optical density at 450 nm using an ELISA reader.

12. The mean of the two duplicates of each sample is taken and the difference of the optical density between coated and uncoated wells is calculated for each sample. The cut-off for positivity is set at five standard deviations above the mean value of a control cohort.

For the detection of IgG subclasses, use subclass specific secondary antibodies (see table below). These are also incubated at 30 min at 37°C on a shaker, with subsequent washing and detection as per the main protocol.

| Anti-human IgG1, HRP conjugated, 1:3000 | Thermo Fisher Scientific | A-10648 |
| --- | --- | --- |
| Anti-human IgG2, HRP conjugated, 1:5000 | Thermo Fisher Scientific | 05-0520 |
| Anti-human IgG3, HRP conjugated, 1:3000 | Thermo Fisher Scientific | 05-3620 |
| Anti-human IgG4, HRP conjugated, 1:2000 | Thermo Fisher Scientific | A-10654 |
