# Supplementary material for: Inter‐Laboratory Validation of Nodal/Paranodal Antibody Testing
Source: J Peripher Nerv Syst. 2025 Jan 29;30(1):e70000. doi: 10.1111/jns.70000 (PMC11780190; doi:10.1111/jns.70000)
Supplement: Supplementary file 2 — Figures S1–S3. Supporting Information Figures S1–S3. [file JNS-30-0-s003.pdf]

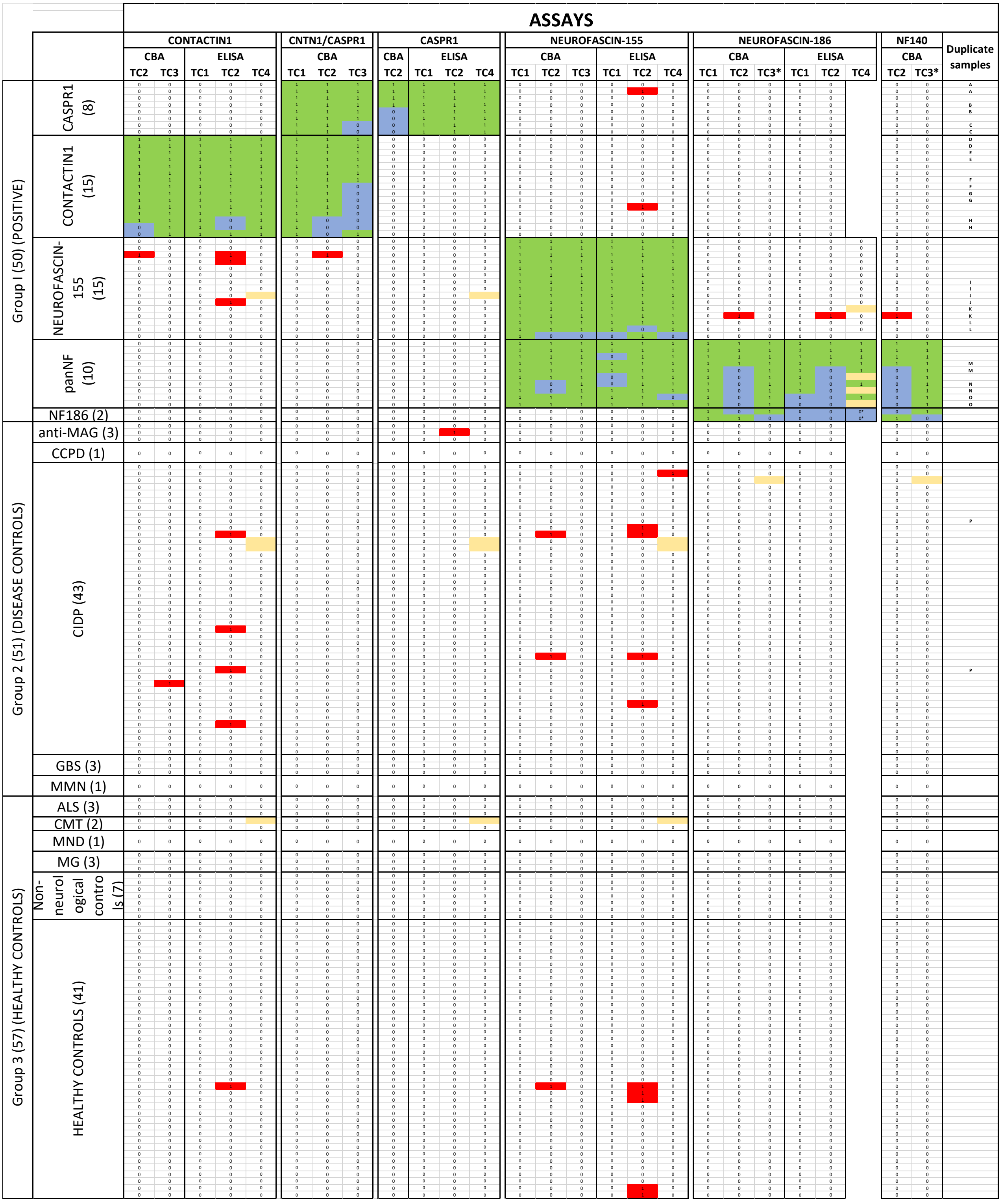


False Positive

True Positive

False Negative

True Negative

Test not performed

**Supplementary Figure 1: Individual assay result for all included samples**

* tests performed after unblinding

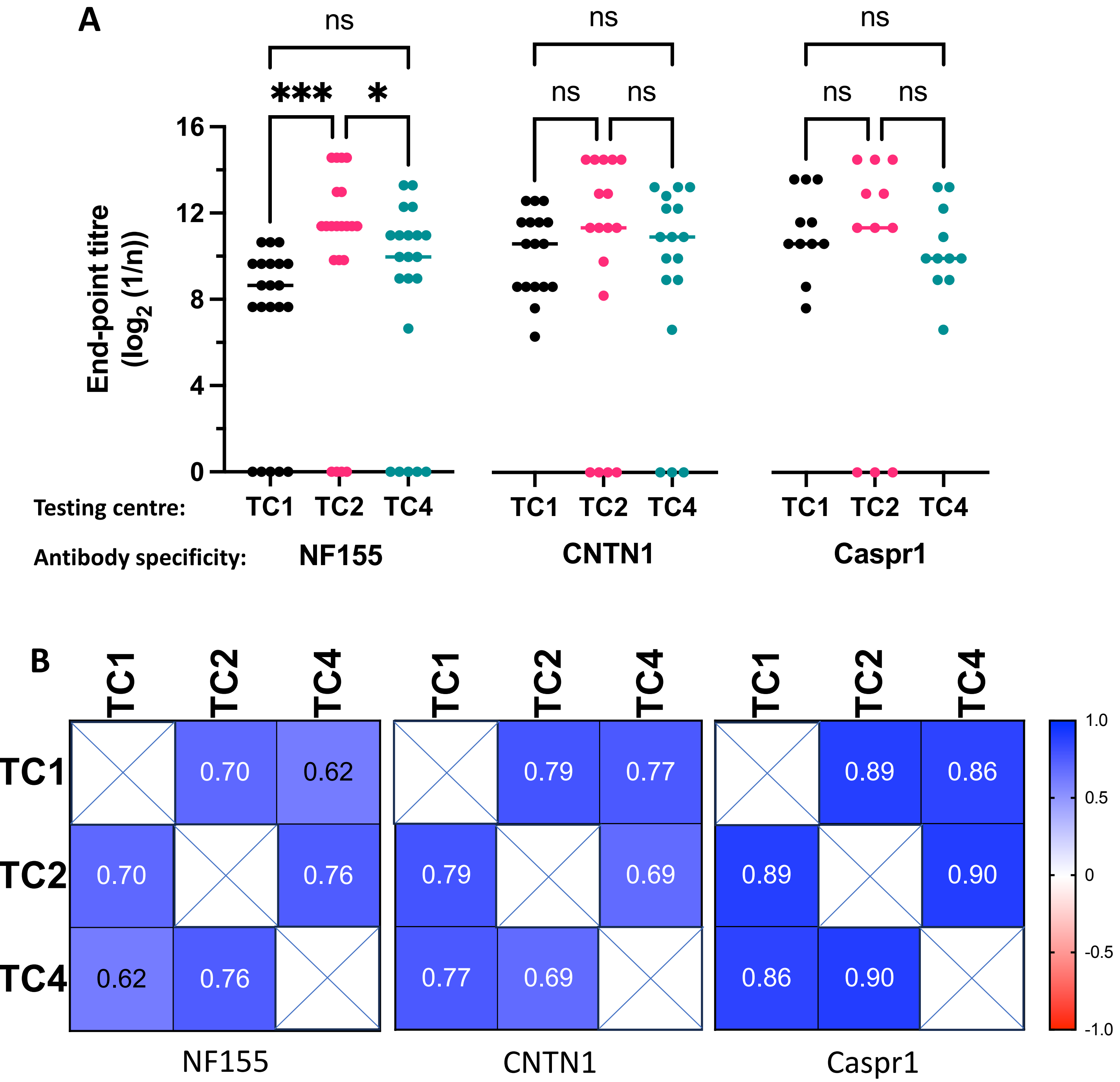


**Supplementary Figure 2: (A)** Distribution of reported end-point titres by testing centre and antibody specificity. *** p<0.001, * p<0.05, ns – not significant (Friedman test). **(B)** Spearman's correlation matrices for end-point titre. (CNTN1 – contactin-1, Caspr1 – contactin associated protein 1, NF – neurofascin)

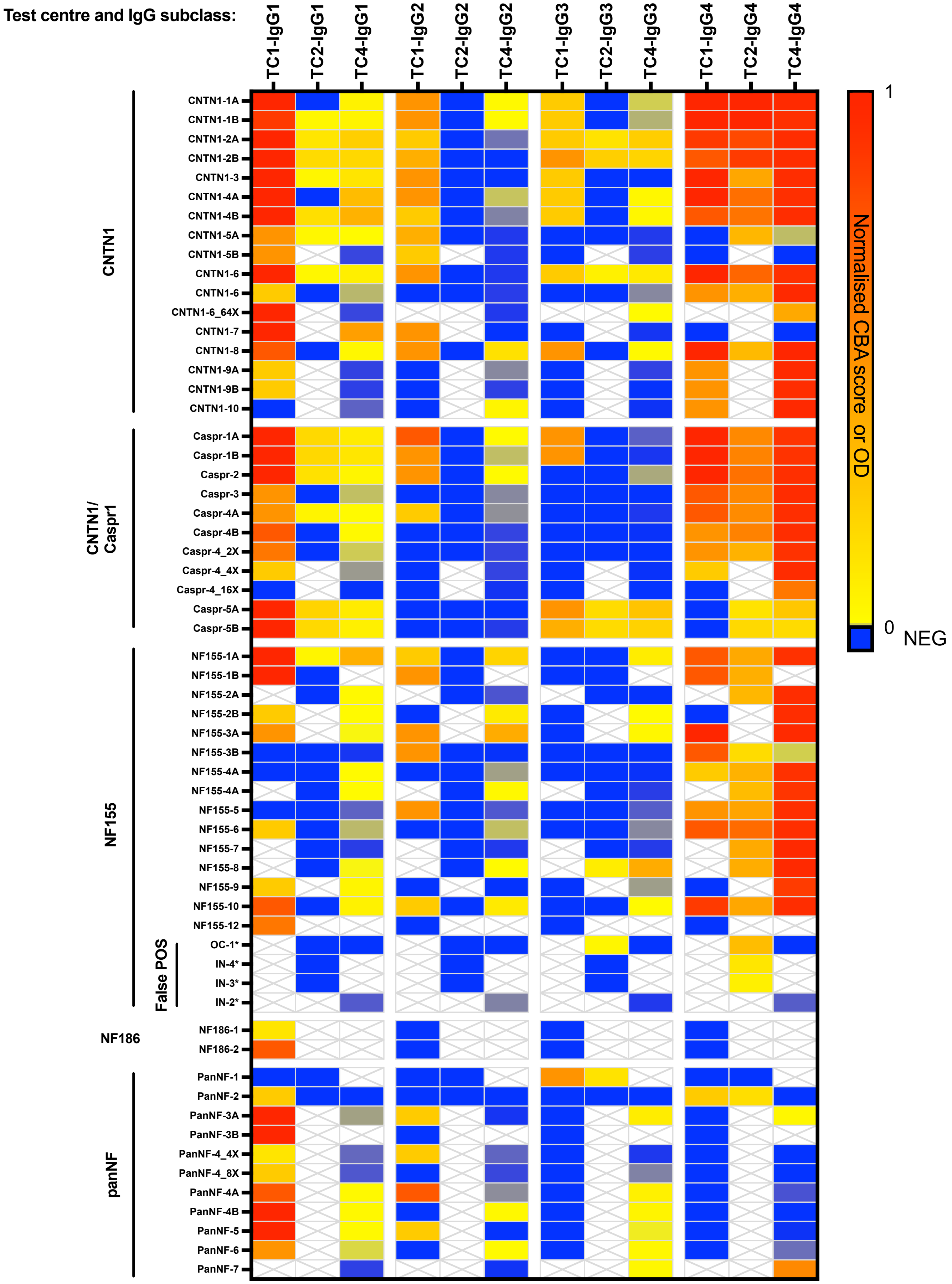


**Supplementary Figure 3: Heatmap of IgG subclasses detected by lab and antigen**

(TC – test centre, CNTN1 – contactin-1, Caspr1 – contactin associated protein 1, NF – neurofascin)

* control samples returning false positive results
