# Supplementary material for: Inter‐Laboratory Validation of Nodal/Paranodal Antibody Testing
Source: J Peripher Nerv Syst. 2025 Jan 29;30(1):e70000. doi: 10.1111/jns.70000 (PMC11780190; doi:10.1111/jns.70000)
Supplement: Supplementary file 3 — Tables A1–A9. Supporting Information Tables A1–A9. [file JNS-30-0-s002.docx]

|  |  | **TC1** | **TC2** | **TC3** | **TC4** | **Individual Totals** | **Group Totals** |
| --- | --- | --- | --- | --- | --- | --- | --- |
| **Group 1:**  **Autoimmune Nodopathy** | **NF-155-Ab+** | **2** | **6 (3)** | **6** | **3 (1)** | **17 (4)** | **52 (16)** |
|  | **NF-186-Ab+** | **2** | **2 (1)** | **0** | **0** | **4 (1)** |  |
|  | **Pan-NF-Ab+** | **4 (1)** | **0** | **0** | **4 (2)** | **8 (3)** |  |
|  | **Contactin-1-Ab+** | **4 (1)** | **5 (2)** | **2** | **4 (2)** | **15 (5)** |  |
|  | **Caspr-1-Ab+** | **2 (1)** | **4 (2)** | **2** | **0** | **8 (3)** |  |
| **Group 2:**  **Other Inflammatory Neuropathies** | **CIDP** | **9 (1)** | **8** | **15** | **11** | **43 (1)** | **51 (1)** |
|  | **GBS** | **0** | **3** | **0** | **0** | **3** |  |
|  | **MAG-Neuropathy** | **3** | **0** | **0** | **0** | **3** |  |
|  | **MMN** | **1** | **0** | **0** | **0** | **1** |  |
|  | **CCPD** | **1** | **0** | **0** | **0** | **1** |  |
| **Group 3:**  **Healthy Controls and Disease Controls** | **Healthy Controls** | **5** | **9** | **15** | **12** | **41** | **57** |
|  | **ALS/MND** | **1** | **3** | **0** | **0** | **4** |  |
|  | **MG** | **0** | **3** | **0** | **0** | **3** |  |
|  | **Genetic neuropathies** | **0** | **2** | **0** | **0** | **2** |  |
|  | **Non-neurological disease** | **7** | **0** | **0** | **0** | **7** |  |
| **Totals** |  | **41 (4)** | **45 (8)** | **40** | **34 (5)** |  | **160 (17)** |

**Table A1: Study samples by submitting lab, disease group and antigen**

Figures in parentheses represent the number of that total which were double aliquoted prior to blinded redistribution to testing labs.

| **Age** | *(median, range)* | 54.5 | (3-79) |
| --- | --- | --- | --- |
| **Male** | *(n, %)* | 26/34 | (76%) |
| **Acute/sub-acute onset** | *(n, %)* | 20/34 | (58%) |
| **Ataxia** | *(n, %)* | 21/33 | (63%) |
| **Tremor** | *(n, %)* | 14/34 | (41%) |
| **Pain** | *(n, %)* | 16/34 | (47%) |
| **Cranial nerve palsy** | *(n, %)* | 15/34 | (44%) |
| **Autonomic features** | *(n, %)* | 7/34 | (20%) |
| **Respiratory compromise** | *(n, %)* | 7/28 | (25%) |
| **Nephrotic Syndrome** | *(n, %)* | 8/24 | (33%) |

**Table A2: Summary demographic and clinical features of group 1 patients**

|  |  | **not used** | **deteriorated** | **no effect** | **partial effect** | **good response** |
| --- | --- | --- | --- | --- | --- | --- |
| **IVIg** | *n* | 3 | 6 | 17 | 5 | 3 |
|  | *%* | 9% | 18% | 50% | 15% | 9% |
| **Steroids** | *n* | 3 | 1 | 10 | 9 | 8 |
|  | *%* | 10% | 3% | 32% | 29% | 26% |
| **Plasma exchange** | *n* | 14 | 0 | 3 | 9 | 6 |
|  | *%* | 44% | 0% | 9% | 28% | 19% |
| **Rituximab** | *n* | 11 | 0 | 3 | 3 | 17 |
|  | *%* | 32% | 0% | 9% | 9% | 50% |

**Table A3: Treatment responses in group 1 patients**

| CASPR1 | | **TC1** | **TC2** | **TC3** | **TC4** |
| --- | --- | --- | --- | --- | --- |
| CBA | CASPR1/CONTACTIN-1 | ✓ | ✓ | ✓ | N/A |
|  | CASPR1 | ✓* | - | N/A | N/A |
|  | CONTACTIN-1 | ✕ | ✕ | ✕ | N/A |
| ELISA | CASPR1 | ✓ | - | N/A | ✓ |
|  | CONTACTIN-1 | ✕ | ✕ | N/A | N/A |
| CONTACTIN-1 | |  |  |  |  |
| CBA | CASPR1/CONTACTIN-1 | ✓ | ✓ | - | N/A |
|  | CONTACTIN-1 | ✓ | ✓ | ✓ | N/A |
| ELISA | CONTACTIN-1 | ✓ | ✓ | N/A | ✓ |
|  | CASPR1 | ✕ | ✕ | N/A | N/A |
| NEUROFASCIN-155 | |  |  |  |  |
| CBA | NF-155 | ✓ | ✓ | ✓ | N/A |
|  | NF-186 | ✕ | ✕ | N/A | N/A |
|  | NF-140 | N/A | N/A | N/A | N/A |
| ELISA | NF-155 | ✓ | ✓ | N/A | ✓ |
|  | NF-186 | ✕ | ✕ | N/A | N/A |
|  | NF-140 | N/A | N/A | N/A | N/A |
| PAN-NEUROFASCIN | |  |  |  |  |
| CBA | NF-155 | ✓ | N/A | N/A | N/A |
|  | NF-186 | ✓ | N/A | N/A | N/A |
|  | NF-140 | N/A | N/A | N/A | N/A |
| ELISA | NF-155 | - | N/A | N/A | ✓ |
|  | NF-186 | - | N/A | N/A | ✓ |
|  | NF-140 | N/A | N/A | N/A | N/A |
| NEUROFASCIN-186 | |  |  |  |  |
| CBA | NF-155 | ✕ | - | N/A | N/A |
|  | NF-186 | ✓ | ✓ | N/A | N/A |
|  | NF-140 | N/A | N/A | N/A | N/A |
| ELISA | NF-155 | ✕ | - | N/A | N/A |
|  | NF-186 | - | ✓ | N/A | ✓ |
|  | NF-140 | N/A | N/A | N/A | N/A |

**Table A4: Approaches to determining overall result and defining antigenic target**

The strategy used to decide on a result for each serum varied between centres. Here the results required for a sample to be considered antibody positive for each antigen at each centre is shown. A ‘✓’ means a positive result was required, N/A means the test was not run, an ‘X’ means a negative result is required and a ‘–’ means the test was run but the result was not used to classify the overall result. *A positive on the CNTN1/Caspr1 dual CBA with negative CNTN1 and Caspr1 results would be termed CNTN1/Caspr1 complex positive by TC1.

**Table A5: Sensitivity, specificity and accuracy for each individual ELISA or CBA**

Red text indicates tests with more than 1 false positive, sensitivity <90%, specificity or accuracy <99%

|  | **Test centre** | | | |
| --- | --- | --- | --- | --- |
| **Repeat titre** | TC1 | TC2 | TC4 | Mean |
| Identical | 5/12 (41.7%) | 5/12 (41.7%) | 4/12 (33.3%) | 38.9% |
| Within 1 dilution step | 10/12 (83.3%) | 9/12 (75%) | 9/12 (75%) | 77.8% |
| Within 2 dilution steps | 10/12 (83.3%) | 12/12 (100%) | 10/12 (83.3%) | 88.9% |

**Table A6: Short-term (intra-) assay variability in end-point titre**

|  | **Test centre** | | | |
| --- | --- | --- | --- | --- |
| **Repeat titre** | TC1 | TC2 | TC4 | Mean |
| Identical | 4/10 (40%) | 3/17 (17.6%) | 3/8 (37.5%) | 31.7% |
| Within 1 dilution step | 7/10 (70%) | 11/17 (64.7%) | 7/8 (87.5%) | 74.1% |
| Within 2 dilution steps | 10/10 (100%) | 15/17 (88.2%) | 8/8 (100%) | 96.1% |

**Table A7: Inter-assay variability in end-point titre**

| **Centre** | **Method** | **Secondary antibodies used** | | | | **Notes** |
| --- | --- | --- | --- | --- | --- | --- |
|  |  | **IgG1** | **IgG2** | **IgG3** | **IgG4** |  |
| TC1 | CBA | Mouse anti-human IgG1 (unconjugated) (I2513, Sigma) | Mouse anti-human IgG2 (unconjugated) (I5365, Sigma) | Mouse anti-human IgG3 (unconjugated) (I7260, Sigma) | Mouse anti-human IgG4 (unconjugated) (I7385, Sigma) | All used at 1:100 followed by goat anti-mouse IgG (H+L) AF488 tertiary at 1:1000 |
| TC2 | ELISA | Mouse anti-human HRP IgG1 (A-10648, Thermo Fisher) | Mouse anti-human HRP IgG2 (MH1722, Thermo Fisher) | Mouse anti-human HRP IgG3 (MH1732, Thermo Fisher) | Mouse antihuman HRP IgG4 (A-10654, Thermo Fisher) | All used at 1:1000 |
| TC4 | ELISA | Mouse anti-human HRP IgG1 (A-10648, Thermo Fisher) | Mouse anti-human HRP IgG2 Fd (05-0520, Thermo Fisher) | Mouse anti-human HRP IgG3 Hinge (05-3620, Thermo Fisher) | Mouse antihuman HRP IgG4 (A-10654, Thermo Fisher) | All used at 1:1000 |

**Table A8: Secondary antibodies used for subclass analysis**

TC1

|  | Reported Result | CBA | ELISA | CBA AND ELISA POSITIVE | CBA OR ELISA POSITIVE |
| --- | --- | --- | --- | --- | --- |
| Sensitivity | 100% | 100% | 96% | 96% | 100% |
| Specificity | 100% | 100% | 99% | 100% | 99% |
| Accuracy | 100% | 100% | 99% | 100% | 99% |

TC2

|  | Reported Result* | CBA | ELISA | CBA AND ELISA POSITIVE | CBA OR ELISA POSITIVE |
| --- | --- | --- | --- | --- | --- |
| Sensitivity | 84% | 86% | 90% | 81% | 96% |
| Specificity | 97% | 97% | 86% | 97% | 86% |
| Accuracy | 96% | 96% | 87% | 96% | 87% |

**Table A9: Effect of multiple assays on sensitivity, specificity and accuracy**

In this study, no combination of assays improved specificity or accuracy compared to use of the single best test alone. *The reported result in TC2 was additionally based on the use of a teased-nerve fibre assay used in 29 samples only.
